# KAML: improving genomic prediction accuracy of complex traits using machine learning determined parameters

**DOI:** 10.1186/s13059-020-02052-w

**Published:** 2020-06-17

**Authors:** Lilin Yin, Haohao Zhang, Xiang Zhou, Xiaohui Yuan, Shuhong Zhao, Xinyun Li, Xiaolei Liu

**Affiliations:** 1 Key Laboratory of Agricultural Animal Genetics, Breeding and Reproduction, Ministry of Education & College of Animal Science and Technology, Huazhong Agricultural University, Wuhan, 430070 Hubei People’s Republic of China; 2 Key Laboratory of Swine Genetics and Breeding, Ministry of Agriculture, Huazhong Agricultural University, Wuhan, 430070 Hubei People’s Republic of China; 3 School of Computer Science and Technology, Wuhan University of Technology, Wuhan, 430070 China; 4 Department of Biostatistics, University of Michigan, Ann Arbor, MI USA; 5 Center for Statistical Genetics, University of Michigan, Ann Arbor, MI USA

## Abstract

Advances in high-throughput sequencing technologies have reduced the cost of genotyping dramatically and led to genomic prediction being widely used in animal and plant breeding, and increasingly in human genetics. Inspired by the efficient computing of linear mixed model and the accurate prediction of Bayesian methods, we propose a machine learning-based method incorporating cross-validation, multiple regression, grid search, and bisection algorithms named KAML that aims to combine the advantages of prediction accuracy with computing efficiency. KAML exhibits higher prediction accuracy than existing methods, and it is available at https://github.com/YinLiLin/KAML.

## Introduction

In the past decade, genome-wide association studies (GWAS) have provided unprecedented insights into the genetic mechanisms of complex traits [1]. With the advances of high-throughput sequencing technologies, the increasing availability of genetic data from existing association studies has led to a growing interest in the selection of traits in livestock and plants, as well as the prediction of disease susceptibility in humans. Over the past 10 years, genomic selection has been applied to several major livestock species and has more than doubled genetic progress in some; it has become an essential tool for livestock breeding programs [2]. For plant breeding, genomic selection is anticipated to facilitate commercialization of improved genotypes at shorter intervals of time than phenotypic selection. For example, the selection cycle for palm oil breeding has been reduced from 19 to 6 years [3, 4]. In humans, genomic prediction for risk of diseases could be utilized to evaluate the effectiveness of disease prevention strategies tailored according to the individual level of projected risk. It is, therefore, no surprise that genomic prediction for risk of diseases will be one of the key tools in the health care of humans in the near future [5, 6].

A commonly used model is known as linear mixed model (LMM), commonly implemented as genomic best linear unbiased prediction (GBLUP), which involves solving mixed model equations (MME) that incorporate the inverse of a genomic similarity matrix, in order to obtain predictions of the corresponding random effect. Its simplest form assumes that all single nucleotide polymorphisms (SNPs) contribute to the heritability of traits, in that all SNPs come from the same one normal distribution [7, 8]. However, this can limit the prediction accuracy of LMM, especially in cases where a trait is controlled by several major genes. To better fit those large effects, alternative Bayesian framework-based methods have been proposed, which include BayesA, BayesB, BayesLASSO, etc. [9, 10]. These methods allow different SNPs to have their own independent variances that follow a specific distribution (e.g., the inverse chi-square distribution). Furthermore, these Bayesian models may assign the SNPs into a mixture of different groups where each group comes from the same normal distribution, which reduces the computational complexity of the model by decreasing the number of parameters to be estimated. For example, BSLMM assumes a mixture of two normal distributions, which allows an additional variance for a subset of SNPs compared with LMM [11]. BayesR assumes a three-component normal mixture together with a group of zero variance [12], which accommodates SNPs with large, medium, small, or no effect. A Bayesian non-parametric model, named Dirichlet process regression (DPR), assigns SNPs into more groups with an assumption of a *x*-component normal mixture where *x* refers to an unlimited number; however, it showed no significant increase in prediction accuracy compared with BayesR [13]. Typically, the unknown parameters of Bayesian models are estimated and optimized by a Markov Chain Monte Carlo (MCMC) procedure. Although the prediction accuracy of Bayesian methods outperforms LMM in the majority of cases [14], the substantial computational burden of the MCMC procedure means analyses take a long time, and also, it can be difficult to choose the right model for any given trait whose genetic architecture could never be known in practice.

Computational efficiency is an important requirement for the application of genomic selection and genomic prediction (GS/GP) in practice, which often precludes the application of Bayesian methods, and has led to LMM becoming one of the most widely used of the GS/GP methods. Moreover, including the most significant SNPs or the verified QTLs as covariates in the LMM improved the prediction accuracy [15, 16], but the phenotypic variance explained (PVE) by those SNPs or QTLs was limited and false positive associations cannot be avoided. Many researchers have attempted to weight the SNPs to construct a trait-specific genomic relationship matrix (Kinship), such that the optimized random effect enables LMM to be as accurate as Bayesian methods [17–19]. However, any noisy SNP may dilute the effects of causal SNPs in only one random effect.

More recently, a LMM methodology that incorporated multiple random effects was proposed. SNPs can be classified into groups using GWAS results or biological information, such as the annotation of SNPs, which include coding, intronic, and intergenic, leading to the simultaneous fitting of separate random effects derived from each of the different groups of SNPs. The LMM with multiple random effects improved the prediction accuracy when the effect size variances differed markedly across the groups [20, 21]. However, the SNPs within each group still contribute equally to each random effect, which potentially limits the prediction accuracy. The principle of SNP classification was too uncontrollable to achieve stable performance among different species [13]. In addition, the efficient and accurate estimation of variance components of many random effects typically represents a significant challenge.

Inspired by the high prediction accuracy of a SNP-weighted strategy and efficient calculation of LMM, here, we present a Kinship-adjusted-multiple-loci (KAML) linear mixed model, which is a flexible modeling framework that generalizes the LMM to accommodate traits with various types of genetic architectures by incorporating pseudo quantitative trait nucleotides (QTNs) as covariates and a SNP-weighted trait-specific Kinship as the variance-covariance assumption corresponding to the random effect term. The selection of pseudo QTNs and SNP weights are optimized by a machine learning procedure combining cross-validation, multiple regression, grid search, and bisection algorithms. Similar to the regular LMM, KAML fits a single random effect and maintains the computing efficiency. Differently, KAML picks up the SNPs with big effects as covariates and simultaneously gives larger weights to SNPs with moderate effects and smaller weights to SNPs with little or no effects as it constructs the Kinship matrix.

We first applied KAML to the seven human diseases that comprise the Wellcome Trust Case Control Consortium (2007) (WTCCC1). Compared with LMM, BSLMM [11], and BayesR [12], KAML performed similarly to the Bayesian methods and significantly outperformed LMM for most of diseases in terms of the prediction accuracy. Taking advantage of parallel computation, the running time of KAML was several orders of magnitude faster than that of BSLMM or BayesR. Afterwards, we evaluated the stability of all the methods mentioned above using data from real cattle, horse, and maize experiments. The results indicated that KAML was the most stable method in terms of the prediction accuracy. Finally, we demonstrated that KAML still performed well when it was assigned prior to calculated model parameters, and this made KAML as efficient as regular LMM, while generating significantly higher prediction accuracy.

## Results

We evaluated KAML using both simulated and real datasets. To assess the prediction accuracy, each dataset was repeatedly split randomly into a reference sample that contained 80% of individuals and a validation sample that contained the remaining 20%. This procedure was repeated 20 times to evaluate the performance of accuracy and stability, but we ensured that the validation sample remained the same for all methods that were compared. The prediction accuracy was quantified with Pearson’s correlation coefficient or AUROC (area under the receiver operator characteristic curve) for continuous traits or binary traits, respectively [22].

The methods that we compared with KAML included (1) the linear mixed model (LMM) referred to as GBLUP, where all SNPs were assumed to contribute equally to the Kinship matrix [23]; (2) Bayesian sparse linear mixed models (BSLMM), which assumed the effects of SNPs followed a mixture of two normal distributions, one with a smaller variance and one with a larger variance, which combined the advantages of both regular LMMs and sparse regression modeling [11]; and (3) BayesR, which assumed that the SNP effects followed a mixture of four zero mean normal distributions with a fixed variance for each mixture component, was flexibly adapted to traits underlying various types of genetic architectures [12]. Note that both BSLMM and BayesR have been demonstrated recently to outperform a range of existing prediction methods; thus, we did not exhaustively include other prediction methods into comparison in our study [13].

For quantifying computing efficiency, we did not personally adjust the settings of all methods and we simply used the defaults. The number of total/burn-in MCMC iterations was 1000,000/100,000 and 50,000/20,000 for BSLMM and BayesR, respectively. The parameter optimization procedure of KAML was sped up using parallel computing, and the computational resources were assigned automatically according to the number of real-time computational tasks, such as the number of SNPs to be tested, population size, and the cross-validation number for optimizing the unknown parameters of KAML. Therefore, the number of threads that was used for speeding up KAML was not constant but varied from 1 to 132 on our server. The computing efficiency of KAML was tested on the Microsoft R Open (MRO, https://mran.microsoft.com/open) platform instead of the base R (https://www.r-project.org/), and multi-threads were assigned automatically to speed up the mathematical calculations by Intel Math Kernel Library (Intel MKL), which included matrix multiply/inverse and matrix decomposition. The computation time results provided a rough guide to the relative computational burden of different methods.

### Simulation studies

First, we demonstrated the flexibility of KAML compared with a regular LMM. To be as realistic as possible, we simulated phenotypes of three scenarios in heritabilities of 0.2, 0.5, and 0.8 with different groups of SNPs corresponding to various genetic architectures that used the genotypes of the WTCCC1 dataset. Phenotypes were simulated by adding the additive genetic effect values and residual effect values as described in previous studies [24–26]. We considered three scenarios of genetic architectures for simulating the additive genetic effects: (1) a large group of 10,000 SNPs with small effects that followed a normal distribution with a mean value of 0 and a variance of 0.005, representing the “polygenic component”; (2) in addition to the polygenic architecture in scenario 1, we added a small group of 10 SNPs with large or moderate effects that followed a normal distribution with a mean value of 0 and a variance of 0.1, representing the “pseudo QTNs *(pQ)*”, where the “polygenic component” and “*pQ*” contributed equally to the genetic variance; and (3) only “*pQ*” in scenario 2 was simulated, which represented the “major genes.” For each combination of heritability and scenario, 20% random selected phenotypes were marked as missing and treated as the validation dataset. The Pearson correlation coefficients between the additive genetic values and predicted values in the validation dataset were calculated, and the mean values of 100 replicates were used to quantify the prediction accuracy.

**Fig. 1:**
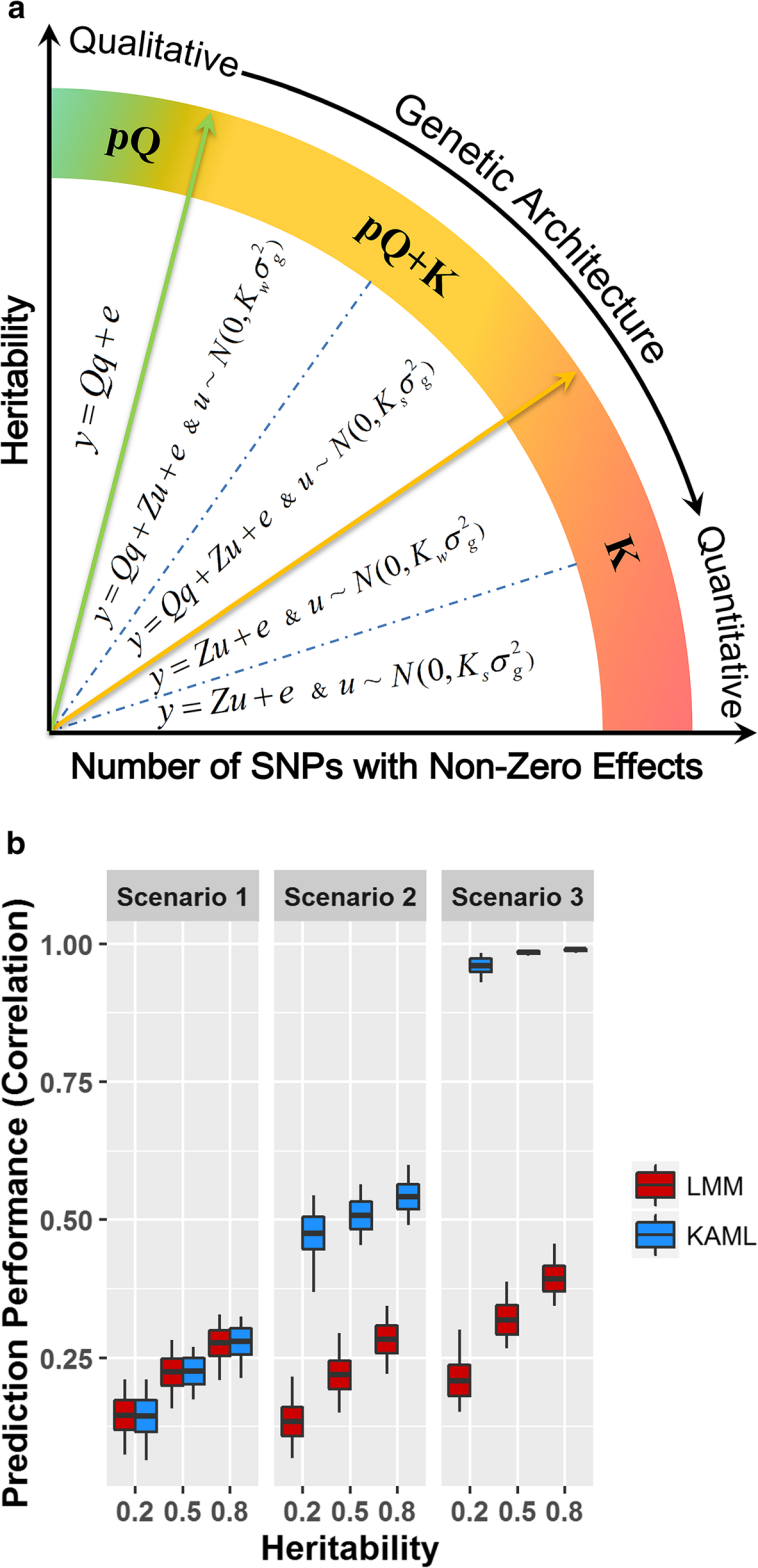
**a** Summary of the achieved models in KAML. Five types of models could be flexibly switched to fit the hypotheses of various types of traits underlying different genetic architectures. The ***pQ*** is the pseudo QTNs, ***K***_***s***_ is the standard Kinship, and ***K***_***w***_ is the SNP-weighted Kinship. **b** The prediction accuracy performances of LMM and KAML on simulated traits. The *x*-axis indexes the simulation scenarios on different levels of heritabilities. The *y*-axis indexes the Pearson correlation coefficients between predicted values and additive genetic effects values in the 20% randomly selected validation dataset for LMM (colored in red) and KAML (colored in blue) across 100 replicates. For each box, the middle line (colored in black) represents the average value, the bottom and top represent the standard deviation, and the top and bottom whiskers represent the maximum and minimum values, respectively

Figure 1a shows the achieved models of KAML that can be applied to various types of qualitative or quantitative traits that underlie different genetic architectures. A total of five types of models can be switched flexibly in KAML: (1) regular LMM (*K*_*s*_), (2) LMM with SNP-weighted Kinship (*K*_*w*_), (3) LMM with pseudo QTNs as covariates and standard Kinship (*pQ+K*_*s*_), (4) LMM with pseudo QTNs as covariates and SNP-weighted Kinship (*pQ+K*_*w*_), or (5) linear model with pseudo QTNs as covariates (*pQ*). Figure 1b summarizes the prediction accuracy performances of LMM and KAML (Additional file 1: Table S1). For scenario 1, the two methods performed similarly with respect to the prediction accuracy, and as we expected, 89%, 69%, and 67% of the KAML models switched to “*K*_*s*_” for the heritability of 0.2, 0.5, and 0.8, respectively (Additional file 1: Table S2), indicating that the hypothesis of LMM was suitable for polygenic traits. Although KAML tended to select the “*pQ+K*” model with an increase in heritability, it made no meaningful difference to the prediction accuracy due to the low proportion of phenotypic variance that was explained by the selected pseudo QTNs. Compared with scenario 1, a small number of SNPs were simulated with large or moderate effects in scenario 2, but the performance of LMM was not improved with the increased number of genetic variants with large effects; accordingly and impressively, there was a significant improvement for KAML when either fitting the optimized pseudo QTNs as covariates or a SNP-weighted Kinship as random effect term, indicating that the strategy of KAML worked correctly. For scenario 3, KAML was far superior to LMM and the results showed that 98%, 96%, and 89% of the models in KAML switched to “*pQ*” for the heritability of 0.2, 0.5, and 0.8, respectively (Additional file 1: Table S2).

To verify its performance, we next compared KAML with GBLUP, BSLMM, and BayesR for three complex traits in three scenarios with heritabilities of 0.2, 0.5, and 0.8, respectively. We simulated the additive genetic effects of the traits that were controlled by 10 SNPs with large effects that were distributed as *N*(0, 0.1), 1000 SNPs with moderate effects that were distributed as *N*(0, 0.01), and 10,000 SNPs with polygenic effects that followed a normal distribution with a mean value of 0 and a variance of 0.005. We implemented the experiments on chromosome 1 of the WTCCC1 dataset with 20 replicates for each scenario [27], and the predictive accuracies are reported in Additional file 1: Table S3 of the supplementary materials. We found that KAML significantly outperformed LMM for all scenarios and performed slightly better than Bayesian models. Furthermore, we evaluated the methods above using a public dataset that was simulated as part of the 16th QTL-MAS Workshop (http://qtl-mas-2012.kassiopeagroup.com/en/dataset.php) [28], and this data has been used widely in comparisons of the performance of alternative methods [19, 29, 30]. The dataset included 1000 reference individuals in each of the first three generations and 1000 validation individuals in generation four. The genotype data had five chromosomes, and each chromosome was simulated with 2000 SNPs. Three traits were simulated and, for each trait, 50 SNPs were randomly selected as QTLs, while their effects followed a gamma distribution (shape parameter = 0.42, scale parameter = 5.4) [19]. The additive genetic effect values for all 4000 individuals were used to assess the prediction accuracy. The results in Table 1 demonstrate the performance of the four methods in terms of prediction accuracy and computing time. For all traits, LMM generated the lowest prediction accuracies compared with the other methods. KAML performed slightly better than BSLMM and BayesR, and the final confirmed models of KAML were all of the form “*pQ+Kw*,” consistent with the simulated genetic architectures. Taking advantage of the parallelized computation, KAML was hundreds of times faster than BSLMM or BayesR.

**Table 1:** Prediction performance in simulated public dataset

Traits/COR^**a**^ (time in h)^**b**^	Methods			
	LMM	BSLMM	BayesR	KAML
**T1**	0.732 (0.002)	0.791 (3.875)	0.795 (0.782)	**0.801** (0.038)
**T2**	0.771 (0.002)	0.831 (4.483)	0.832 (0.714)	**0.843** (0.043)
**T3**	0.758 (0.002)	0.827 (4.721)	0.832 (0.740)	**0.832** (0.039)
Average	0.754	0.816	0.820	0.825

COR^a^: The Pearson correlation coefficient between predicted values and additive genetic effects values. The reference dataset included 3000 individuals and 1000 validation individuals

(Time in h)^b^: The computing time is recorded in hours

By choosing the most appropriate prediction model, pseudo QTNs, and optimizing a SNP-weighted Kinship in a cross-validation process, KAML had the potential to change the model hypothesis according to the data feature automatically, and it can be adapted to a wide range of traits underlying various types of genetic architectures. Furthermore, KAML could significantly shorten the computational effort without sacrificing prediction accuracy. In practice, we will never know the real genetic architecture in advance, which makes the stable performance of KAML very appealing.

### WTCCC1 data

In addition to the simulated data, KAML was also evaluated using the real published data from human, cattle, horse, and maize experiments. We firstly assessed the performance of KAML for seven disease traits from the Wellcome Trust Case Control Consortium (WTCCC1, https://www.wtccc.org.uk/) [31]. When comparing the performance of methods on binary disease traits (cases 1, controls 0), we treated the phenotypic records as continuous variables following previous studies [11, 21, 32], and the predicted values were then considered to be the probability of being the case. The WTCCC1 dataset included about 1400 cases and 2938 shared controls, and all individuals were genotyped for about 450,000 SNPs. The seven diseases were bipolar disorder (BD), coronary artery disease (CAD), Crohn’s disease (CD), hypertension (HT), rheumatoid arthritis (RA), type 1 diabetes (T1D), and type 2 diabetes (T2D).

The information from the GWAS results was used to select the pseudo QTNs and to optimize the SNP weights in KAML. Various statistical models have been utilized to detect the trait-associated SNPs [24, 26, 33, 34], and results varied in different datasets. The rank and the significance of SNPs in the results obtained by different statistical models may potentially affect the processes of selecting pseudo QTNs and optimizing Kinship in KAML. Therefore, we compared the prediction accuracy of KAML when using a general linear model (GLM) or a mixed linear model (MLM) for the GWAS procedure; the Manhattan plot from GLM can be found at Additional file 1: Fig. S1. Benefiting from the combination strategies of grid search, bisection method, and cross-validation, we found that there was no significant difference between those two association test models in any of the WTCCC1 datasets (Additional file 1: Table S4). Consequently, we recommended the GLM because it generated similar prediction accuracy, but ran much faster than MLM. In addition, we obtained a slight improvement in prediction accuracy when we increased the number of cross-validation replicates, but this resulted in an increased computational burden (Additional file 1: Table S4). It is often necessary to balance prediction accuracy performance and computational efficiency accordingly.

We next compared the prediction accuracy performance of KAML with that of LMM, BSLMM, and BayesR. Table 2 reports the prediction accuracy performances of those models using WTCCC1 datasets. Here, we conducted KAML using GLM and a 4*5 cross-validation procedure (5-fold cross-validation repeated four times) to estimate the unknown parameters for traits. We found that KAML performed as well as BSLMM and BayesR for all diseases, and it outperformed LMM for most diseases, which was consistent with the simulation. In particular, KAML outperformed BSLMM and BayesR for three diseases (CD, RA, and T1D) where a small number of relatively strong associations were identified in the original study [31]. Moreover, KAML, BSLMM, and BayesR performed slightly worse than LMM for HT and BD, as we expected, and no pseudo QTNs were selected as covariates in KAML for those two diseases (Additional file 1: Table S7), which indicated that both traits were polygenic, and all markers contributed very little to the traits and matched the marker effect size distribution hypothesis of LMM, which was consistent with the situation that no significant associations were detected (Supplemental Fig. 1). However, we noted that none of the methods could be competitive for all traits, since the genetic architecture was more complex than implied by the models [35].

**Table 2:** Comparison of prediction accuracy performances of LMM, BSLMM, BayesR, and KAML by using seven case/control diseases in the WTCCC1 dataset

Traits/AUROC	Methods			
	LMM	BSLMM	BayesR	KAML
**CAD**	0.586 (0.0043)	0.599 (0.0041)	**0.602 (0.0039)**	0.600 (0.0041)
**HT**	**0.597 (0.0038)**	0.596 (0.0038)	0.596 (0.0041)	0.594 (0.0038)
**T2D**	0.600 (0.0032)	0.618 (0.0032)	**0.620 (0.0034)**	0.618 (0.0031)
**BD**	0.641 (0.0033)	0.641 (0.0034)	**0.647 (0.0033)**	0.638 (0.0035)
**CD**	0.628 (0.0039)	0.669 (0.0041)	0.668 (0.0046)	**0.669 (0.0039)**
**RA**	0.614 (0.0034)	0.704 (0.0032)	0.708 (0.0033)	**0.717 (0.0037)**
**T1D**	0.646 (0.0045)	0.858 (0.0019)	0.861 (0.0018)	**0.862 (0.0019)**
Average	0.616	0.669	0.672	0.671

Prediction accuracy performance was measured by the area under the ROC curve (AUROC). For prediction assessment, total samples were split to two subsets: 80% of the samples were used as the reference dataset and 20% were used as the validation dataset; the procedure was repeated 20 times; and the mean AUROC values and standard deviations of each trait are shown in the table

Table 3 reports the average computing time of LMM, BSLMM, BayesR, and KAML in hours. LMM was the most efficient model due to its simple hypothesis for all traits. Benefiting from the parallel computing design, KAML was computationally very efficient, and the computing time was several orders of magnitude lower than BSLMM and BayesR. The computing time for both BSLMM and BayesR varied with the complexity of the architectures of traits, polygenic traits required much more time to reach convergence. Because only two groups of SNP effects were assumed in BSLMM instead of four in BayesR, as well as an improved parameter sampling strategy for fast convergence, BSLMM was faster than BayesR for most of the traits.

**Table 3:** The computing performance tests of LMM, BSLMM, BayesR, and KAML methods by using the WTCCC1 dataset

Traits/time (hours)	Methods			
	LMM	BSLMM	BayesR	KAML
**CAD**	0.01 (0.00)	13.78 (2.55)	44.93 (2.45)	0.30 (0.01)
**HT**	0.01 (0.00)	27.15 (5.25)	42.83 (2.46)	0.32 (0.02)
**T2D**	0.01 (0.00)	38.11 (6.15)	45.85 (2.17)	0.32 (0.01)
**BD**	0.01 (0.00)	23.5 (6.26)	40.21 (2.26)	0.35 (0.03)
**CD**	0.01 (0.00)	56.55 (5.02)	32.87 (0.80)	0.35 (0.01)
**RA**	0.01 (0.00)	3.85 (0.27)	33.22 (0.78)	0.40 (0.00)
**T1D**	0.01 (0.00)	5.98 (0.27)	34.23 (1.02)	0.4 (0.00)
Average	0.01	24.13	39.16	0.35

Computing performance tests were conducted in a Red Hat Enterprise Linux sever with 2.20 GHz Intel(R) Xeon(R) 132 CPUs E7-8880 v4, and 2 TB memory. The computing time records and the standard deviations are described in Table 2. The computing performances of BSLMM and BayesR methods were tested using their default settings

### Cattle/horse/maize

**Fig. 2:**
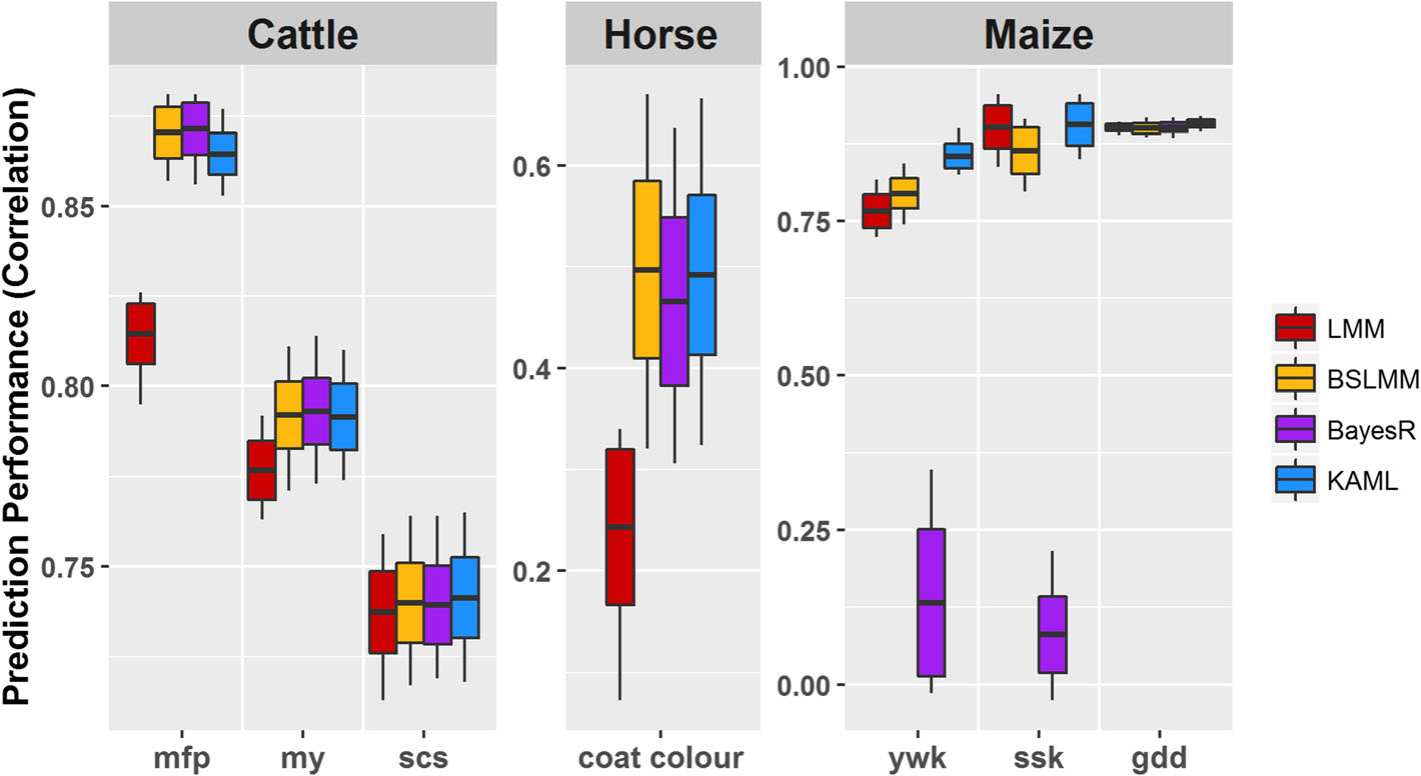
Comparison of prediction accuracy performances of LMM (red), BSLMM (yellow), BayesR (violet), and KAML (blue) in cattle, horse, and maize datasets. The prediction accuracy performance of each method was measured by the correlation method, which is the average Pearson correlation between predicted values and phenotypic values of 20 replicates in the validation subset. In each replicate, the dataset was randomly split into a reference subset containing 80% of individuals and a validation subset containing the remaining 20%. For each boxplot, the middle line represents the average value, the bottom and top are the standard deviation, and the upper and lower ends of each box represent the maximum and minimum, respectively

In addition to the WTCCC1 dataset, we assessed the prediction accuracy performance of LMM, BSLMM, BayesR, and KAML using datasets of multiple species, including animal (cattle and horse) and plant (maize) populations. Details about the population size and available SNPs were described in the Data Quality Control section. Seven traits were used for performance tests: four of which were quantitative traits, including milk fat percentage (mfp), milk yield (my), somatic cell score (scs), and growing degree days (gdd), and three were qualitative traits, including coat color, yellow or white kernels (ywk), and sweet or starchy kernels (ssk). Considering the situation that inbreeding may increase false positive associations in GWAS due to cryptic relationships among individuals (e.g., a population includes a parent with a large number of progenies in the population [36]), the GWAS model was switched to MLM in KAML for animal and plant datasets, and a 1*5 cross-validation procedure was used to reduce computational effort. Figure 2 shows the prediction accuracy performances of the four models (Additional file 1: Table S5).

**Fig. 3:**
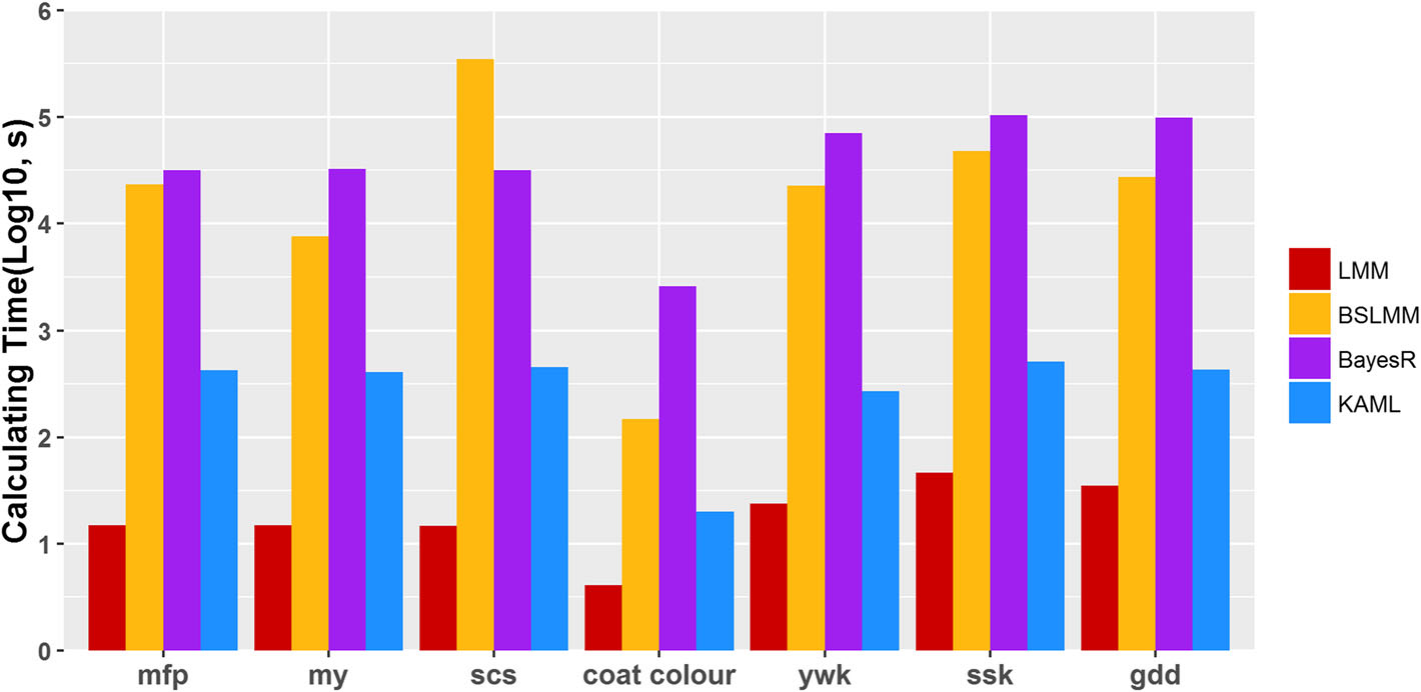
The comparison of computing performances (in seconds) of LMM (red), BSLMM (yellow), BayesR (violet), and KAML (blue) for cattle, horse, and maize datasets. The *y*-axis represents the computing time in log_10_ scale. Computing performance tests were performed in a Red Hat Enterprise Linux sever with 2.20 GHz Intel(R) Xeon(R) 132CPUs E7-8880 v4, and 2 TB memory. The computing time records are corresponding to the experiments described in Fig. 2. The computing performances of BSLMM and BayesR methods were tested using their default settings

For the cattle data, BayesR outperformed the other methods for traits mfp and my, while KAML performed best for trait scs. For the horse data, as the provided phenotype from the publishers were already coded as 1, 2, 3, and 4 for corresponding colors believed to result from three known coat color loci that included *MC1R* (chestnut), *STX17* (gray), and *ASIP* (agouti, black/bay) [37], we used the original phenotype as a continuous trait to run all software directly to make fair comparisons. From the results, we found that both KAML and BSLMM performed better than BayesR, and most of the selected models in KAML were “*pQ*” (Additional file 1: Table S7), suggesting that the coat color was influenced by a small number of SNPs with large effects, which mismatched the hypotheses of the four marker effect distributions of BayesR, and this maybe a reason that BayesR took a long time to reach MCMC convergence and performed much slower than BSLMM for this trait (Fig. 3). For maize data, KAML performed the best among the four methods. Strangely, BayesR performed abnormally for traits ywk and ssk when using the default number of MCMC iterations.

Our previous study showed that when the marker density was increased, MCMC-based methods required more iterations to reach convergence and to obtain reliable prediction values. In order to figure out the problem, we designed a series of gradients of total iteration numbers to track trends in predictive performance. As shown in Additional file 1: Fig. S2, when the total number of iterations reached 200,000, BayesR tended to perform as accurately as BSLMM, suggesting that BayesR required more MCMC iterations for the traits ywk and ssk. On the contrary, BSLMM performed well at a low number of MCMC steps. For these three traits, the performance declined continuously for BSLMM with an increase in total iteration number, that because BSLMM is the combination of the LMM and BVSR [38, 39], and the MCMC procedure of BSLMM for estimating the unknown parameters started at the values derived from LMM, so its performance was similar to LMM when the total MCMC iteration number was small, the dropped accuracy is caused by the mismatch of genetic architecture between the hypothesis and the analyzed traits. Therefore, it is a challenge for MCMC-based methods to determine the appropriate number of total MCMC iterations to ensure an accurate and stable prediction performance for traits with unknown genetic architectures. On the whole, KAML performed stably for all traits of multiple species, indicating that the hypothesis of KAML had the potential to be adapted to a wide range of genetic architectures. Additionally, KAML was roughly 100–300 times faster than BSLMM or BayesR (Fig. 3 and Additional file 1: Table S6). Moreover, KAML could be accelerated by more computing resources.

### Predicting with pre-optimized parameters for KAML

The most time-consuming part of KMAL is for the estimation of variance components in the cross-validation procedure. Although the procedure can be sped up by parallel computation, it is still a challenge when dealing with big datasets with limited computational resources. In fact, it is not necessary for KAML to re-optimize the weights of markers repeatedly for the same trait when the dataset reaches a “sufficient” size, since the contributions of common SNPs could be captured easily without a very large population [23]. To verify this assumption, we implemented a further experiment using all the datasets mentioned above on KAML and compared its prediction performance with LMM. We randomly selected half of the total individuals only once, and then we ran KAML to obtain all model parameters (*model type*, *pseudo QTNs*, *α*, and *β*, for details, see the “Methods” section) by a default setting of KAML for each trait. Then the optimized parameters were directly used in KAML to evaluate the prediction accuracy performance using the same replicates above. Note that an extra GWAS within reference subset for each replicate needed to be done to calculate the Kinship matrix with the known *α* and *β*, but the time-consuming cross-validation procedure was excluded. Compared with optimizing the unknown parameters adaptively every time, this saved a great deal of time.

**Fig. 4:**
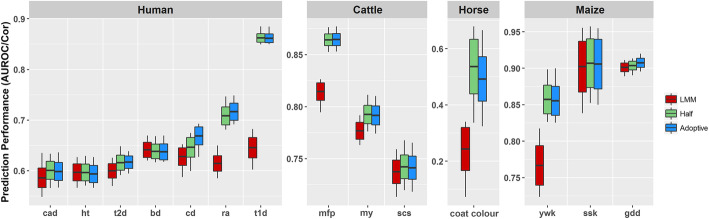
The comparison of prediction accuracy performances of LMM and KAML. The performance was measured by the AUROC and Pearson correlation for the datasets of human and other species, respectively. ***Half*** represents that KAML randomly selected only half of the total individuals to optimize the model parameters; ***Adaptive*** represents running KAML on the entire dataset, and the parameters were optimized for each replicate. For each boxplot, the middle line represents the average value, the bottom and top are the standard deviation, and the upper and lower ends of each box represent the maximum and minimum, respectively

Figure 4 reports the predictive performances of LMM and KAML (***Half***, ***Adaptive***). We found that the ***Half*** performed even better than ***Adaptive*** in some traits (coat color, ywk) but performed similarly in most cases (Additional file 1: Table S8). It was quite clear that both ***Half*** and ***Adaptive*** performed significantly better than LMM. This result indicated that it was not necessary to re-optimize the unknown parameters for KAML when the population size was increased slightly. Especially in animal breeding, the unknown parameters of KAML could be renewed weekly or even annually, which makes the computational complexity of KAML closer to LMM and could save much time in practice (Additional file 1: Table S9).

### Predicting by integrating KAML into SSBLUP

It is always difficult to genotype all individuals with phenotypic records for a large population. Therefore, a single-step BLUP (SSBLUP) [40, 41] method was developed to utilize the pedigree information, genotype information, and phenotype information simultaneously to estimate individual genetic values. It has become the most widely used model in livestock breeding programs due to its high predictive performance [42]. The SNP-weighted Kinship matrix from KAML can be directly integrated with SSBLUP to construct a trait-specific mixture relationship matrix for all available individuals. Using the simulated data in the 16th QTL-MAS Workshop, which contained pedigree information, we compared the prediction accuracy performances of PBLUP (with the relationship matrix derived from pedigree information only), LMM (with the relationship matrix derived from genotype information only), SSBLUP (with the relationship matrix derived from information of both pedigree and genotype), KAML (with SNP-weighted Kinship matrix derived from genotype information only and incorporating the *pQs* as covariates), and SSKAML (with weighted Kinship matrix derived from information of both pedigree and genotype) methods. The comparative results are shown in Table 4 and indicated that the SSKAML outperformed SSBLUP and LMM methods for both non-genotyped and genotyped individuals. It was noted that the KAML outperformed SSKAML for the genotyped individuals in terms of prediction accuracy; one potential reason was that the *pQs* were incorporated as covariates in the KAML model, which is unachievable for SSKAML due to the absence of genotypes for non-genotyped individuals. The results highlighted the potential of KAML in the application of genomic selection.

**Table 4:** The comparison of prediction performances on the 16th QTL-MAS Workshop dataset

Traits/accuracy	Non-genotyped (***n*** = 100)			Genotyped (***n*** = 1000)				
	PBLUP	SSBLUP	SSKAML	PBLUP	LMM	SSBLUP	KAML	SSKAML
T1	0.7823	0.8648	0.8819	0.4549	0.7317	0.7322	0.8011	0.7969
T2	0.7954	0.8726	0.8901	0.5331	0.7708	0.7707	0.8433	0.8378
T3	0.7401	0.8238	0.8418	0.4704	0.7581	0.7579	0.8324	0.8262

P*BLUP* pedigree information-based BLUP method, *LMM* genomic information-based BLUP method, *SSBLUP* single-step BLUP method. For SSBLUP, the weight of pedigree-based relationship matrix for genotyped individuals was 0.05. The diagonal and off-diagonal of both pedigree and genomic relationship matrices were adjusted to the same scale

## Discussion and conclusion

Because of the flexible hypotheses, Bayesian methods are well known for their high predictive accuracy. A large number of unknown parameters, including thousands or even millions of marker effects, should be estimated. The complex posterior distributions and computational complexity of traditional multiple integrals limited the implementation of Bayesian methods. The problem was solved after the MCMC method was introduced to Bayesian statistics. However, the effects of millions of markers can need a large number of MCMC iterations to reach convergence of the posterior means and the iterations cannot easily be accelerated by parallel computing, which limits the practical application of Bayesian methods. In contrast, the LMM efficiently predicts individual genetic values using the relationship information, and all markers are assumed in a sense to contribute equally to the construction of Kinship matrix. The marker contribution can also be weighted under the LMM framework, but this makes the modified LMM face the same problem as Bayesian methods—a large number of unknown parameters need to be estimated. KAML adopted a machine learning procedure combining cross-validation, multiple regression, grid search, and bisection algorithms for unknown parameter estimation to solve the problem and reduce thousands or millions of unknown parameters to two (*α* and *β*), which can be combined with the marker *p* value from GWAS results to weight the marker contributions. Compared with MCMC, the machine learning procedure can be accelerated with parallel computing and is much more efficient.

Marker effects and *p* values were reported to be used to weight the marker contributions in modified LMM to improve the prediction accuracy [43]. In KAML, we used the *p* value, which represented how significant the genetic marker was associated with the objective trait. It should be pointed out that there are two potential concerns for integrating GWAS results to improve the predictive accuracy. The first is that LD between genetic markers and causal mutations are usually different between the training subsets and individuals to be predicted. In our implementation, the genetic markers were tested *x* times, where *x* is the number of significant detections in a bootstrap strategy-based GWAS from the cross-validation procedure. The bootstrap strategy resampled the individuals a number of times to reduce the effect of population-specific LD to some extent, which filtered the pseudo QTNs that were far from the causal mutations. The problem could be avoided to some extent by the increasing marker density and number of repeats in the cross-validation procedure. The second problem was that the *p* values were different when the genetic markers were tested by different GWAS methods. It is challenging to know how to assign appropriate weights of genetic markers using the *p* values from different GWAS methods to obtain a robust predictive accuracy. In KAML, two parameters, which included the base value of logarithmic function of *p* values and the percentage of top significant genetic markers to be weighted, were optimized in a combined method of grid search and bisection to eliminate to some extent the effect of the *p* values in terms of absolute value. However, this does not mean that GWAS methods do not affect the prediction accuracy. A GWAS method with higher statistical power for a given false discovery rate will certainly provide a better *p* value rank of genetic markers to improve the prediction accuracy compared to using an under powered GWAS.

In either selection of pseudo QTNs or construction of the SNP-weighted Kinship, the unknown parameters were optimized by the machine learning procedure combining cross-validation, multiple regression, grid search, and bisection algorithms. The number of folds (*s*) and repeats (*v*) in the cross-validation procedure influence the prediction accuracy. It was difficult to determine *s* and *v*, and appropriate sets were related to the size of reference population and the genetic architecture of the objective trait. In this study, we found that more repeats improved prediction accuracy of KAML, but significantly increased computing time. Therefore, we recommend setting the number of repeats according to the data size and computing resources. With the help of the cross-validation strategy of machine learning procedure, KAML can switch automatically to five types of models (Fig. 1a). Hence, it has the potential to adjust the model hypothesis based on features of the data, and it can be adapted to a wide range of genetic architectures. With the advantage of an efficient parallelized parameter optimization procedure, KAML is computationally efficient and roughly hundreds of times faster than BSLMM and BayesR methods, as well as other MCMC-based methods, while generating similar or better predictive performance.

The usage of pedigree information can sometimes be limited in plants and humans due to incomplete pedigree records or small family sizes and in plants due to novel modes of reproduction, such that the single-step strategy is currently only used widely in livestock breeding. We compared the integrated prediction accuracy of combining SSBLUP with KAML. In practical breeding, SSKAML with pre-estimated parameters led to a higher prediction accuracy compared with SSBLUP and maintained the advantage of computing speed. It highlighted the great potential of KAML in its application of genomic selection.

Functional annotations, reported QTLs, Encode, etc. can be used as prior knowledge to weight the contributions of genetic markers in the prediction model. With the increasing number of publications reporting research for complex diseases, prior knowledge will be enriched by advanced statistical analyses and integrating this information would potentially improve the prediction accuracy. However, it is difficult to evaluate the reliability of prior knowledge, and directly incorporating the information in the prediction model is of high risk. Therefore, we recommend utilizing prior knowledge in the GWAS model instead of directly increasing the weights of genetic markers. The machine learning-based parameter optimization procedure would help to assess the top significant genetic markers of GWAS results and to evaluate the prior knowledge indirectly, and this enables KAML to provide a robust prediction accuracy performance.

In conclusion, aiming to exploit the advantages of both prediction accuracy and computing speed, we proposed a machine learning-based method combining cross-validation, multiple regression, grid search, and bisection algorithms named KAML. The new method incorporates pseudo QTNs as covariates and a trait-specific random effect term under the LMM framework and provides a flexible assumption to accommodate traits of various genetic architectures. We demonstrated the reliability and robustness of KAML by both rigorous simulations and real data analyses. We recommend the use of KAML in practice because of its high prediction performance and advanced computing efficiency. The KAML method is implemented in an easy-to-use software tool that is freely available to the public.

## Methods

KAML is a flexible model that extends LMM by integrating pseudo QTNs as covariates and an optimized trait-specific random effect. All unknown parameters are optimized by machine learning procedure combining cross-validation, multiple regression, grid search, and bisection algorithms. The pseudo QTNs are derived from a multiple regression model-based selection procedure, and random effect relevant unknown parameters (e.g., the weights of SNP markers) are optimized by combining grid search and bisection algorithm. Here, we firstly provide a brief overview of LMM, and then give the description of theoretical differences between LMM and KAML.

### Linear mixed model

The standard LMM assumes that *y*, which is the phenotypic records for *n* individuals, follows a normal distribution, and its variance is influenced by effects from both heredity and environment. The LMM model can be described as:

1 $$ y= Xb+ Z\mu +\mathrm{with}\ \mu \sim N\left(0,K{\sigma}_g^2\right)\ \mathrm{and}\ e\sim N\left(0,I{\sigma}_e^2\right), $$

where *b* is a vector of the estimated effects of the fixed covariates with the corresponding coefficient matrix *X*; *μ* is a vector of random effects, which represents the individual genetic values with the corresponding variance-covariance matrix *K* that is also defined as genomic relationship matrix; *e* is the vector of residual effects; *I* is an identity matrix; and ${\sigma}_g^2$ and ${\sigma}_e^2$ are the estimated genetic variance and residual variance, respectively. The genetic relationship between individuals *i* and *j* is calculated as: [23]

2 $$ {K}_{ij}=\frac{1}{m}{\sum}_{k=1}^m\frac{\left({M}_{ik-}2{p}_k\right)\ \left({M}_{jk-}2{p}_k\right)}{2{p}_k\left(1-{p}_k\right)}, $$

where *m* is the number of markers, *M* is the numerical genotype matrix (AA, AB, and BB genotypes are coded as 0, 1, and 2, respectively), and *p* is the frequency of the coded allele. The genetic values of all individuals including observed and non-observed phenotypic records can be derived by the following equation:

3 $$ \mu =K{Z}^T{V}^{-1}\left(y- Xb\right) $$

where *b* = (*X*^*T*^*V*^−1^*X*)^−1^(*X*^*T*^*V*^−1^*y*) and $$ V= ZK{Z}^T{\sigma}_g^2+I{\sigma}_e^2 $$.

### Machine learning determined parameter optimizations (KAML)

The LMM assumes that all available SNPs contribute equally to the Kinship matrix. This limits its prediction accuracy, especially in cases that the objective traits are controlled by several major genes. Therefore, KAML extends Eq. 1 to include *n* covariates *Q*_*1*_, *Q*_*2*_, … *, Q*_*n*_, which are derived from a multiple regression model-based selection procedure, and a SNP-weighted Kinship (*K*_*w*_), which is optimized by the combination of grid search and bisection algorithm:

4 $$ y= Xb+ Qq+Z{\mu}^{\ast }+e\ \mathrm{with}\ {\mu}^{\ast}\sim N\left(0,{K}_w{\sigma}_g^2\right)\ \mathrm{and}\ e\sim N\left(0,I{\sigma}_e^2\right), $$

KAML firstly integrates some major SNPs into the model as covariates to capture some of the genetic variance, then optimizes a SNP-weighted Kinship for the random effect to make a better explanation for the remaining genetic variance. The derivation of weighting SNPs in constructing Kinship matrix is described in the paper published by Su et al. [43] and can be formulated as follows:

5 $$ {K}_{w_{ij}}=\frac{1}{m}{\sum}_{k=1}^m\frac{\left({M}_{ik}-2{p}_k\right){\xi}_k\left({M}_{jk}-2{p}_k\right)}{2{p}_k\left(1-{p}_k\right)} $$

where *ξ*_*k*_ is the weight of *k*th SNP. In contrast to previous studies, we propose a more flexible and robust weighting strategy with fewer unknown parameters to be estimated; the weight *ξ*_*k*_ is derived from the following equation:

6 $$ {\xi}_k\mid \left(\alpha, \beta \right)\sim \left\{\begin{array}{c}1\kern4.75em ;\kern0.5em 1-\beta \\ {}1+{\mathit{\log}}_{\alpha }{P}_{m\beta}-{\mathit{\log}}_{\alpha }{P}_k;\kern1.50em \beta \end{array}\right. $$

where *P* is the ordered *p* values of all SNPs from GWAS result, *α* is the base value of logarithmic function, and *β* is the percentage of top significant SNPs to be weighted. If *β* equals 0, Eq. 5 will switch to Eq. 2. It can be simplified as:

7 $$ {K}_{w_{ij}}={K}_{ij}+\frac{1}{m}{\sum}_{k=1}^{m\beta}\frac{\left({M}_{ik}-2{p}_k\right)\left({\mathit{\log}}_{\alpha }{P}_{m\beta}-{\mathit{\log}}_{\alpha }{P}_k\right)\left({M}_{jk}-2{p}_k\right)}{2{p}_k\left(1-{p}_k\right)} $$

As shown above, this could be computed on-the-fly with a subset instead of all SNPs. Unlike LMM, the estimated genetic values of KAML include two parts: the first part is the fixed effect and the second part is the random effect:

8 $$ {\mu}^{\ast }= Qq+{K}_w{Z}^T{V}^{-1}\left(y- Xb- Qq\right) $$

As described above, the key steps of KAML are identifying the SNPs with large effects and estimating the unknown parameters *α* and *β* efficiently. KAML achieves those two goals by a machine learning procedure, which includes multiple regression for pseudo QTN selection and grid search, bisection algorithms for trait-specific weighted Kinship matrix, and cross-validation, which is considered to be one of the most powerful and accurate methods in parameter estimation [44]. Compared with MCMC procedures which are commonly used to estimate parameter in Bayesian methods, the machine learning procedure can be readily accelerated by parallel computation. Hence, the entire procedure of KAML includes two stages: (1) a training stage, which is used to obtain the optimal parameters from the reference population including all individuals with phenotypic records, the reference population set is partitioned for cross-fold internal validation to tune the parameters in KAML, and (2) a prediction stage, which predicts the genetic value of each individual without phenotype by the optimal parameters obtained from the training stage.

#### Genome-wide association study

A genome-wide association study is the prevailing method for detecting candidate genes underlying traits and has successfully detected substantial numbers of variants that are associated with human diseases and agricultural economic traits. For the trait to be predicted, GWAS is a powerful tool to capture its genetic architecture, as the *p* values provide information for the downstream model optimization of KAML. Define *s* as the repeat number and *v* as the fold number of cross-validation procedure. For each replicate, the individuals with non-missing phenotype are equally divided into *v* groups, GWAS is conducted *v* times by using the data of randomly combined *v* − 1 groups, and the data of the left-out group is used as validation subset for the parameter optimization.

#### The pseudo QTNs derived by multiple regression

It is very sensitive to integrate the pseudo QTNs (*pQ*) as covariates, because a false positive can significantly decrease the prediction accuracy; therefore, we cautiously constitute a rigorous strategy to reduce the risk of selecting false positives. First, we order the *p* values from small to large. Second, filter the ordered SNPs by linkage disequilibrium (LD) at a threshold of 0.3. Third, simultaneously pick up the top *n* pseudo QTNs to ensure valuable signals of multiple peaks can be captured. Finally, we repeat the first three steps in a *s* ∗ *v* cross-validation procedure. The bootstrap strategy is used to measure the robustness of SNP association [45]. GWAS is conducted *s* ∗ *v* times in total, and the SNPs that are counted more than *s* ∗ *v* ∗ 90% times are considered as *pQ* for downstream multiple regression analysis. It should be noted that the number of *pQ* (*n*) is never known; a smaller number may lead to losing *pQ* and a greater number would result in more calculation burden. Fortunately, the setting of *n* slightly affects the prediction accuracy as the weights of lost *pQ* could be enhanced in the SNP-weighted procedure, and the default setting of *n* is set to 15.

The LD between genetic markers and causal mutations may be different in the reference and validation populations, and this will increase the risk of false positive associations due to limited marker density. In order to reduce this type of false positive associations, *pQs* are added as covariates in LMM one by one without replacement and validated to determine whether they can help the model to improve the prediction accuracy in a cross-validation procedure. The *pQs* are simultaneously validated in three types of models: “*K*” (LMM), “*pQ+K*” (*pQ* are incorporated as covariates in LMM), and “*pQ*” (*pQ* is incorporated as covariates in GLM), and a series of models are generated. The reliability of each *pQ* is validated by its average prediction accuracy in a *s* ∗ *v* cross-validation procedure, and the equation can be written as:

9 $$ {\overline{A}}_{q_i}=\raisebox{1ex}{$\sum \limits_{k=1}^{s\ast v}{A}_{q_{ik}}$}\!\left/ \!\raisebox{-1ex}{$s\ast v$}\right. $$

where ${A}_{q_{ik}}$ is the accuracy of *i*th *pQ* for *k*th cross-validation; it should be pointed out that two methods, including Pearson correlation and area under the receiver operator characteristic curve (AUROC), are implemented in KAML to compute the prediction accuracy; KAML will automatically switch to use AUROC when the phenotype is coded in two levels by 0 and 1, and to use Pearson correlation for other cases. The AUROC is calculated under the guidance of the paper published by Wray et al. in 2010 [22]. It is defined as follows:

10 $$ \mathrm{AUROC}=\frac{1}{N_{d\prime }}\left({\overline{r}}_d-\frac{N_d}{2}-\frac{1}{2}\right) $$

where *N*_*d*_ and *N*_*d*′_ are the numbers of diseased and not diseased individuals, respectively, and ${\overline{r}}_d$ is the mean rank of the diseased individuals. ${\overline{A}}_{q_i}$ is the average accuracy of *i*th *pQ*. Let ${\overline{A}}_0$ refer to the average prediction accuracy of LMM. The maximum prediction accuracy values of three types of models are compared to decide the final prediction model type for the application stage. The *pQ* under the condition of ${\overline{A}}_{q_{i-1}}<{\overline{A}}_{q_i}\&{\overline{A}}_0<{\overline{A}}_{q_i}$ will be selected as the effective *pQ* in the application stage.

#### The SNP-weighted Kinship optimized by grid search and bisection algorithms

**Fig. 5:**
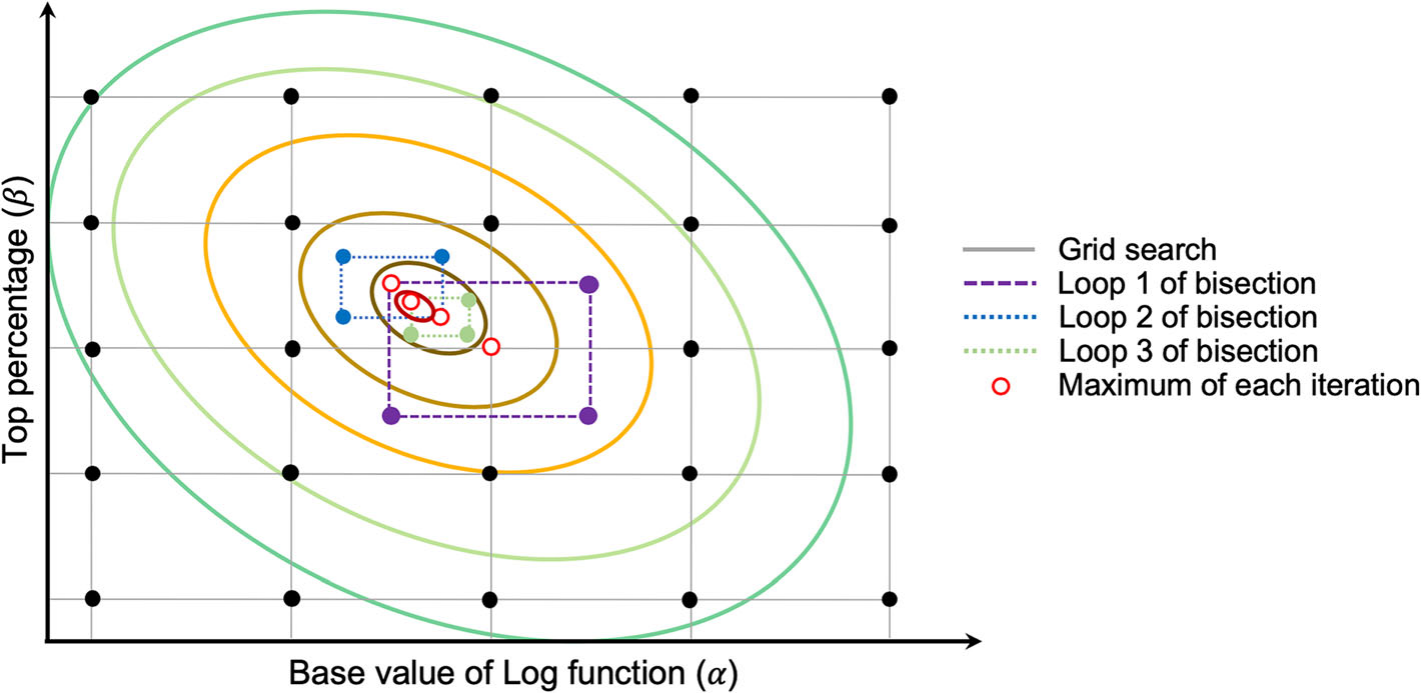
Illustration of grid search and bisection algorithm in SNP-weighted Kinship optimization. The ovals are contour line, and the smallest circle at the center is the optimum combination of *α* and *β* in theory. A combination strategy of grid search and bisection methods are used to search the optimum combination of *α* and *β*. In order to avoid multiple peaks around the maximum value in each iteration, four points are set on both sides (*x*- and *y*-axes) instead of one. The iteration procedure will be stopped until the maximum number of iterations is reached or the difference of prediction accuracy values between the last two iterations is less than a pre-set threshold, e.g., 10e^−5^

If the confirmed model of multiple regression is not “*pQ*,” a combination strategy of grid search and bisection methods, which are used to derive a SNP-weighted Kinship, will be conducted. To start the grid search procedure, start values should be provided for unknown parameters *α* and *β*, respectively. By default, KAML provides the start values of *α*_1_, *α*_2_, …, *α*_*n*1_ and *β*_1_, *β*_2_, …, *β*_*n*2_ as (1.01, 1.11, *e*, 10) and (0.0001, 0.001, 0.01, 0.1), respectively, where *e* is the base of natural logarithm. The SNP-weighted Kinship can be constructed following Eq. 6 by using the corresponding GWAS results from cross-validation, and then the prediction accuracy performances of combinations ${A}_{\alpha_1{\beta}_1},{A}_{\alpha_1{\beta}_2},\dots, {A}_{\alpha_2{\beta}_1},{A}_{\alpha_2{\beta}_2},\dots, {A}_{\alpha_{n1}{\beta}_{n2}}$ will be recorded for *s* ∗ *v* times in the cross-validation procedure, and the average prediction accuracy of different combinations of *α* and *β* can be calculated as:

11 $$ {\overline{A}}_{\alpha_i{\beta}_j}=\raisebox{1ex}{$\sum \limits_{k=1}^{s\ast v}{A}_{\alpha_{ik}{\beta}_{jk}}$}\!\left/ \!\raisebox{-1ex}{$s\ast v$}\right. $$

where ${A}_{\alpha_{ik}{\beta}_{jk}}$ is the accuracy of the combination of *i*th *α* and *j*th *β* at the *k*th cross-validation. ${\overline{A}}_{\alpha_i{\beta}_j}$ is the average accuracy of the combination of *α* and *β*. The combinations of *α* and *β* are the intersection points of black solid lines shown in Fig. 5.

The point with the maximum prediction accuracy value ${\overline{A}}_{\alpha \beta}$ in the grid search procedure (the hollow ring in Fig. 5) is assigned to be the start of bisection procedure. In order to avoid multiple peaks around ${\overline{A}}_{\alpha \beta}$, we reset two levels at the midpoint on both sides of *α* and *β*. Let ${\phi}_{\alpha_1}^{\ast },{\phi}_{\alpha_2}^{\ast }$ and ${\phi}_{\beta_1}^{\ast },{\phi}_{\beta_2}^{\ast }$ be the reset values for *α* and *β*, respectively (the intersection of dotted line in Fig. 5):

12 $$ \left({\phi}_{\alpha}^{\ast },{\phi}_{\beta}^{\ast}\right)\in {\phi}^{\ast}\sim f(x)=\left\{\begin{array}{c}U\left(\frac{\phi_i}{2},\frac{\phi_i+{\phi}_{i+1}}{2}\right)\kern3.25em ;i=1\kern1.25em \\ {}U\left(\frac{\phi_{i-1}+{\phi}_i}{2},\frac{\phi_i+{\phi}_{i+1}}{2}\right)\kern1.25em ;1<i<n\\ {}U\left(\frac{\phi_{i-1}+{\phi}_i}{2},\frac{3{\phi}_i-{\phi}_{i-1}}{2}\right)\kern1.25em ;i=n\ \end{array}\right. $$

where *U* is the uniform distribution and *i* is the iteration number of bisection procedure. Similarly, we predict the individuals in validation dataset to get the average accuracy ${\overline{A}}_{\phi_{\alpha_1{\beta}_1}^{\ast }},{\overline{A}}_{\phi_{\alpha_1{\beta}_2}^{\ast }},{\overline{A}}_{\phi_{\alpha_2{\beta}_1}^{\ast }},{\overline{A}}_{\phi_{\alpha_2{\beta}_2}^{\ast }}$ by Eq. 8 and we select the maximum value ${\overline{A}}_{\phi_{\alpha \beta}^{\ast }}$. Subsequently, stepping to the next iteration with the newly updated ${\phi}_{\alpha_1}^{\ast },{\phi}_{\alpha_2}^{\ast }$ and ${\phi}_{\beta_1}^{\ast },{\phi}_{\beta_2}^{\ast }$, which are defined by Eq. 12. The iteration procedure will continue until the maximum number of iterations is reached or the difference of prediction accuracy values between the last two iterations is less than a pre-set threshold, e.g., 10e^−5^.

Once the iteration procedure stops, we compare the ${\overline{A}}_{\phi_{\alpha \beta}^{\ast }}$ and ${\overline{A}}_0$ to determine whether the SNP-weighted Kinship could help to increase the prediction accuracy. If ${\overline{A}}_{\phi_{\alpha \beta}^{\ast }}$ < ${\overline{A}}_0$, the optimizations will be given up, and KAML uses the standard Kinship without weighting any SNPs. On the contrary, if the optimizations are validated to be effective, then KAML will construct the SNP-weighted Kinship using the optimum combination of *α* and *β*.

#### Prediction for application stage

If the final prediction model is selected to be “*pQ*,” the individual genetic values will be directly predicted by LM with the selected *pQ*. If not, an extra GWAS needs to be conducted for the entire reference population, or we merge the bootstrap GWAS results of cross-validation by mean, then a SNP-weighted Kinship matrix can be computed using the optimum combination of *α*, *β*, and the GWAS results, which finally will be applied to predict the individual genetic values.

### Dataset

The WTCCC1 data includes approximately 14,000 cases from seven common diseases and 2938 shared controls, and all individuals were genotyped at about 450,000 SNPs. Following previous analyses of the datasets [12, 21], we removed SNPs using PLINK [46], with either minor allele frequency (MAF) < 0.01, or genotype call rate (CR) < 0.95, or *p* value < 0.05 from Hardy-Weinberg equilibrium (HWE) test. After being filtered, 1868 cases and 373,369 SNPs of bipolar disorder (BD), 1926 cases and 372,541 SNPs of coronary artery disease (CAD), 1748 cases and 374,113 SNPs of Crohn’s disease (CD), 1952 cases and 373,338 SNPs of hypertension (HT), 1860 cases and 373,056 SNPs of rheumatoid arthritis (RA), 1963 cases and 372,964 SNPs of type 1 diabetes (T1D), and 1924 cases and 373,149 SNPs of type 2 diabetes (T2D) remained for prediction performance tests.

The cattle dataset was a German Holstein genomic prediction population comprising 5024 bulls [19]. All bulls were genotyped with the Illumina Bovine SNP50 Beadchip [47]. After removing the SNPs with either HWE *p* value < 10^−4^ or CR < 0.95 or MAF < 0.01, a total of 42,551 SNPs remained for the downstream analysis. The estimated breeding values of three traits were available and used in this study: milk fat percentage (mfp), milk yield (my), and somatic cell score (scs). The three traits may represent three types of genetic architectures composed of (1) one or several major genes and a large number of loci with small effects (mfp), (2) a few moderate effect loci and many small effect loci (my), and (3) many loci with small effects (scs).

The maize data consisted of 2279 inbred accessions and three traits, including two case/control traits: yellow or white kernels (ywk) and sweet or starchy kernels (ssk), and one quantitative trait: growing degree days (gdd). A total of 681,257 SNPs across all maize lines were obtained with genotyping by sequencing (GBS) [48]. After removing SNPs with either MAF < 0.01 or CR < 0.95, 314 controls, 1281 cases, and 631,413 SNPs for ywk; 2490 controls, 141 cases, and 633,754 SNPs for ssk; 2279 individuals and 631,674 SNPs for GDD remained in this study [49].

The horse data included 14 domestic horse breeds and 18 evolutionarily related species. In total, 480 horses were genotyped with a designed ~ 54,000 SNP assay. A total of 50,621 SNPs was available after removing the SNPs with MAF < 0.01, CR < 0.90, and *p* value < 0.001 from the HWE test. The trait was coat color, which contained 4 levels of classified variables, and it was previously reported to be regulated by a major gene on chromosome 3 [37].

## Supplementary information

**Additional file 1.** Tables S1–S9 and Figs. S1–S3.

**Additional file 2.** Review history.

## Data Availability

This study makes use of data generated by the Wellcome Trust Case Control Consortium (WTCCC). A full list of the investigators who contributed to the generation of the WTCCC data is available from http://www.wtccc.org.uk/, where the dataset can be publicly accessed. Funding for the WTCCC project was provided by the Wellcome Trust under award 076113 and 085475. The following datasets can be directly downloaded from the links below: QTL-MAS-2012: https://figshare.com/articles/QTL-MAS-2012/12336866; Horse: https://figshare.com/articles/Horse_dataset/12336773; Cattle: https://www.g3journal.org/content/5/4/615.supplemental; and Maize: https://datacommons.cyverse.org/browse/iplant/home/shared/panzea. The source code of KAML is available at a DOI-assigning repository Zenodo (10.5281/zenodo.3757055) [50] and at GitHub (https://github.com/YinLiLin/KAML) under the GNU General Public License v3.0.
