# Supplementary material for: KAML: improving genomic prediction accuracy of complex traits using machine learning determined parameters
Source: Genome Biol. 2020 Jun 17;21:146. doi: 10.1186/s13059-020-02052-w (PMC7386246; doi:10.1186/s13059-020-02052-w)
Supplement: Supplementary file 1 — Additional file 1. Tables S1–S9 and Figs. S1–S3. [file 13059_2020_2052_MOESM1_ESM.docx]

**KAML: improving genomic prediction accuracy of complex traits using machine learning determined parameters**

Supplementary Material

Lilin Yin^1,2^, Haohao Zhang^3^, Xiang Zhou^4,5^, Xiaohui Yuan^3^, Shuhong Zhao^1,2^, Xinyun Li^1,2,*^, and Xiaolei Liu^1,2,*^

^1^ Key Laboratory of Agricultural Animal Genetics, Breeding and Reproduction, Ministry of Education & College of Animal Science and Technology, Huazhong Agricultural University, Wuhan, Hubei, 430070, PR China;

^2^ Key Laboratory of Swine Genetics and Breeding, Ministry of Agriculture, Huazhong Agricultural University, Wuhan 430070, Hubei, PR China;

^3^ School of Computer Science and Technology, Wuhan University of Technology, Wuhan 430070, China;

^4^ Department of Biostatistics, University of Michigan, Ann Arbor, MI, USA;

^5^ Center for Statistical Genetics, University of Michigan, Ann Arbor, MI, USA.

^*^ Corresponding author. Email: xiaoleiliu@mail.hzau.edu.cn; xyli@mail.hzau.edu.cn

Table S1: Comparison of prediction performances of LMM and KAML in various simulated scenarios.

| **Methods** | **Scenario 1** | |  | **Scenario 2** | |  | **Scenario 3** | |
| --- | --- | --- | --- | --- | --- | --- | --- | --- |
| **Heritability** | **LMM** | **KAML** |  | **LMM** | **KAML** |  | **LMM** | **KAML** |
| 0.2 | 0.146 | 0.144 |  | 0.134 | 0.476 |  | 0.209 | 0.961 |
| 0.5 | 0.224 | 0.226 |  | 0.219 | 0.508 |  | 0.319 | 0.985 |
| 0.8 | 0.277 | 0.280 |  | 0.284 | 0.542 |  | 0.393 | 0.989 |
| **Average** | **0.216** | **0.217** |  | **0.221** | **0.509** |  | **0.307** | **0.978** |

The values report the average prediction accuracy performances (correlation) of 100 replicates for each situation. Each situation represents a combination of model, scenario, and heritability.

Table S2: The counts of selected models in the model parameter optimization procedure of KAML in various simulated scenarios.

| **Models** | **Scenario 1** | | |  | **Scenario 2** | | |  | **Scenario 3** | | |
| --- | --- | --- | --- | --- | --- | --- | --- | --- | --- | --- | --- |
| **Heritability** | ***pQ*** | ***pQ+K*** | ***K*** |  | ***pQ*** | ***pQ+K*** | ***K*** |  | ***pQ*** | ***pQ+K*** | ***K*** |
| 0.2 | 0 | 11 | 89 |  | 15 | 85 | 0 |  | 98 | 2 | 0 |
| 0.5 | 0 | 31 | 69 |  | 0 | 100 | 0 |  | 96 | 4 | 0 |
| 0.8 | 0 | 33 | 67 |  | 0 | 100 | 0 |  | 89 | 11 | 0 |

In each situation, the value reports the number of selected models (“*pQ”, “pQ+K”, “K”*) for 100 replicates in the model parameter optimization procedure. Each situation represents a combination of model, scenario, and heritability. “*pQ”*: Linear Model with pseudo QTNs as covariates; *“pQ+K”*: LMM with pseudo QTNs as covariates and standard (or weighted) Kinship; *“K”*: LMM with standard (or weighted) Kinship.

Table S3: Comparison of the prediction performances of LMM, BSLMM, BayesR, and KAML methods on simulated traits using human dataset.

| **Heritability** | **Methods** | | | |
| --- | --- | --- | --- | --- |
|  | **LMM** | **BSLMM** | **BayesR** | **KAML** |
| 0.2 | 0.4312 | 0.5632 | 0.5510 | 0.5680 |
| 0.5 | 0.5980 | 0.6815 | 0.6724 | 0.6814 |
| 0.8 | 0.7167 | 0.7722 | 0.7680 | 0.7728 |
| **Average** | **0.5820** | **0.6723** | **0.6638** | **0.6741** |

Prediction accuracy was measured using Pearson correlation coefficients between the predicted values and the simulated additive genetic effect values. The average Pearson correlation coefficients of each scenario are shown on the basis of 20 replicates in the table.

Table S4: Prediction performances of KAML using different GWAS models and a variable number of cross validation folds in dataset of human diseases.

| **Traits**  **AUROC/Time(h)** | **KMAL** | | | |
| --- | --- | --- | --- | --- |
|  | **GWAS** | **s1_c5** | **s2_c5** | **s4_c5** |
| CAD | GLM | 0.596/0.12 | 0.599/0.17 | 0.600/0.3 |
|  | MLM | 0.596/0.41 | 0.599/0.64 | 0.600/1.18 |
| HT | GLM | 0.590/0.14 | 0.593/0.18 | 0.594/0.32 |
|  | MLM | 0.591/0.43 | 0.594/0.64 | 0.594/1.23 |
| T2D | GLM | 0.614/0.13 | 0.617/0.18 | 0.618/0.32 |
|  | MLM | 0.615/0.42 | 0.616/0.66 | 0.618/1.19 |
| BD | GLM | 0.636/0.12 | 0.638/0.19 | 0.638/0.35 |
|  | MLM | 0.636/0.41 | 0.637/0.63 | 0.638/1.20 |
| CD | GLM | 0.665/0.13 | 0.669/0.19 | 0.669/0.35 |
|  | MLM | 0.665/0.39 | 0.668/0.62 | 0.668/1.14 |
| RA | GLM | 0.715/0.15 | 0.716/0.21 | 0.717/0.4 |
|  | MLM | 0.715/0.42 | 0.716/0.68 | 0.716/1.22 |
| T1D | GLM | 0.861/0.15 | 0.861/0.22 | 0.862/0.4 |
|  | MLM | 0.863/0.42 | 0.863/0.7 | 0.863/1.28 |

Prediction performance was measured by the AUROC method. For the prediction assessment of each trait, 20 replicates were carried out, and the dataset was randomly split into a reference subset that contained 80% of individuals and a validation subset that contained the remaining 20% in each replicate. The “GLM” and “MLM” represent a linear model and a mixed linear model, respectively. “s1_c5” means to repeat the 5-folds cross validation procedure 1 time, and so on.

Table S5: Comparison of the prediction performances of LMM, BSLMM, BayesR, and KAML methods in datasets of multiple species.

| **Species** | **Traits** | **Methods** | | | |
| --- | --- | --- | --- | --- | --- |
|  |  | **LMM** | **BSLMM** | **BayesR** | **KAML** |
| Cattle | mfp | 0.8145 | 0.8704 | 0.8713 | 0.8645 |
|  | my | 0.7766 | 0.7921 | 0.7931 | 0.7916 |
|  | scs | 0.7372 | 0.7397 | 0.7393 | 0.7412 |
| Horse | coat color | 0.2432 | 0.4971 | 0.4655 | 0.4919 |
| Maize | ywk | 0.7667 | 0.7951 | 0.1321 | 0.8553 |
|  | ssk | 0.9021 | 0.8638 | 0.0800 | 0.9058 |
|  | gdd | 0.9012 | 0.9001 | 0.9019 | 0.9074 |

Prediction accuracy was measured using Pearson correlation coefficients between the predicted values and actual phenotypic records (Horse and Maize) or estimated breeding values (Cattle). For the prediction assessment of each trait, 20 replicates were carried out, and the dataset was randomly split into a reference subset that contained 80% of individuals and a validation subset that contained the remaining 20% in each replicate.

Table S6: Computing performance tests (h) of LMM, BSLMM, BayesR, and KAML methods using datasets of multiple species.

| **Species** | **Traits** | **Methods** | | | |
| --- | --- | --- | --- | --- | --- |
|  |  | **LMM** | **BSLMM** | **BayesR** | **KAML** |
| Cattle | mfp | 0.0041 | 6.7118 | 9.0806 | 0.1177 |
|  | my | 0.0041 | 2.1986 | 9.3223 | 0.1124 |
|  | scs | 0.0041 | 102.2675 | 9.0467 | 0.1263 |
| Horse | coat color | 0.0011 | 0.0407 | 0.7375 | 0.0057 |
| Maize | ywk | 0.0066 | 6.3501 | 19.8108 | 0.0743 |
|  | ssk | 0.0129 | 13.6788 | 29.1575 | 0.1416 |
|  | gdd | 0.0098 | 8.4913 | 27.6100 | 0.1188 |

The values are reported in hours. The computing performance tests were conducted in a Red Hat Enterprise Linux server with 2.20 GHz Intel(R) Xeon(R) 132 CPUs E7-8880 v4 and 2 TB memory.

Table S7: Counts of selected models in the model parameter optimization procedure of KAML using datasets of multiple species.

| **Species** | **Traits** | **KAML** | | | | |
| --- | --- | --- | --- | --- | --- | --- |
|  |  | ***pQ*** | ***pQ+Kw*** | ***pQ+Ks*** | ***Kw*** | ***Ks*** |
| Human | cad | 0 | 12 | 2 | 6 | 0 |
|  | ht | 0 | 0 | 0 | 13 | 7 |
|  | t2d | 0 | 13 | 0 | 7 | 0 |
|  | bd | 0 | 0 | 2 | 15 | 3 |
|  | cd | 0 | 20 | 0 | 0 | 0 |
|  | ra | 0 | 20 | 0 | 0 | 0 |
|  | t1d | 0 | 20 | 0 | 0 | 0 |
| Cattle | mfp | 0 | 20 | 0 | 0 | 0 |
|  | my | 0 | 20 | 0 | 0 | 0 |
|  | scs | 0 | 20 | 0 | 0 | 0 |
| Horse | coat colour | 18 | 1 | 1 | 0 | 0 |
| Maize | ywk | 0 | 20 | 0 | 0 | 0 |
|  | ssk | 0 | 9 | 0 | 11 | 0 |
|  | gdd | 0 | 20 | 0 | 0 | 0 |

The ***pQ*** is the pseudo QTNs, ***Ks*** is the standard Kinship, and ***Kw*** is the SNP-weighted Kinship. The five models in KAML are (1) regular LMM (“*Ks*”), (2) LMM with SNP-weighted Kinship (“*Kw*”), (3) LMM with pseudo QTNs as covariates and standard Kinship (“*pQ+Ks*”), (4) LMM with pseudo QTNs as covariates and SNP-weighted Kinship (“*pQ+Kw*”), and (5) Linear Model with pseudo QTNs as covariates (“*pQ*”).

Table S8: Comparison of prediction performances of LMM and KAML with/without optimized parameters in various datasets.

| **Species** | **Traits** |  | **Methods** |  |
| --- | --- | --- | --- | --- |
|  |  | **LMM** | **Half** | **Adaptive** |
| Human | cad | 0.5859 | 0.6008 | 0.6000 |
|  | ht | 0.5967 | 0.5965 | 0.5936 |
|  | t2d | 0.5997 | 0.6158 | 0.6176 |
|  | bd | 0.6411 | 0.6381 | 0.6378 |
|  | cd | 0.6275 | 0.6462 | 0.6686 |
|  | ra | 0.6142 | 0.7083 | 0.7165 |
|  | t1d | 0.6455 | 0.8620 | 0.8619 |
| Cattle | mfp | 0.8145 | 0.8642 | 0.8645 |
|  | my | 0.7766 | 0.7924 | 0.7916 |
|  | scs | 0.7372 | 0.7421 | 0.7412 |
| Horse | coat colour | 0.2432 | 0.5360 | 0.4919 |
| Maize | ywk | 0.7667 | 0.8572 | 0.8553 |
|  | ssk | 0.9021 | 0.9068 | 0.9058 |
|  | gdd | 0.9012 | 0.9035 | 0.9074 |

The prediction performances were measured by the AUROC method and Pearson correlation coefficients between the predicted values and actual phenotypic records (or estimated breeding values) for datasets of humans and other species. ***Half*** represents the KAML model with only half of the total individuals selected randomly to optimize model parameters; ***Adaptive*** represents running KAML on the entire dataset, and the parameters were optimized for each replicate. In each replicate, ***Half*** and ***Adaptive*** shared the same individuals to be predicted.

Table S9: Computing performance tests (sec) of LMM and KAML with/without optimized parameters in various datasets.

| **Species** | **Traits** |  | **Methods** |  |
| --- | --- | --- | --- | --- |
|  |  | **LMM** | **Half** | **Adaptive** |
| Human | cad | 52 | 104 | 1,139 |
|  | ht | 51 | 103 | 1,138 |
|  | t2d | 50 | 110 | 1,220 |
|  | bd | 50 | 103 | 1,487 |
|  | cd | 48 | 98 | 1,543 |
|  | ra | 51 | 110 | 1,779 |
|  | t1d | 50 | 113 | 1,499 |
| Cattle | mfp | 15 | 85 | 424 |
|  | my | 15 | 82 | 405 |
|  | scs | 15 | 84 | 455 |
| Horse | coat colour | 4 | 1 | 20 |
| Maize | ywk | 24 | 73 | 267 |
|  | ssk | 46 | 138 | 510 |
|  | gdd | 35 | 119 | 428 |

The values are reported in seconds. The computing performance tests were conducted in a Red Hat Enterprise Linux sever with 2.20 GHz Intel(R) Xeon(R) 132 CPUs E7-8880 v4, and 2 TB memory.

Fig. S1. The Manhattan plots of GWAS results from human, cattle, horse, and maize datasets.


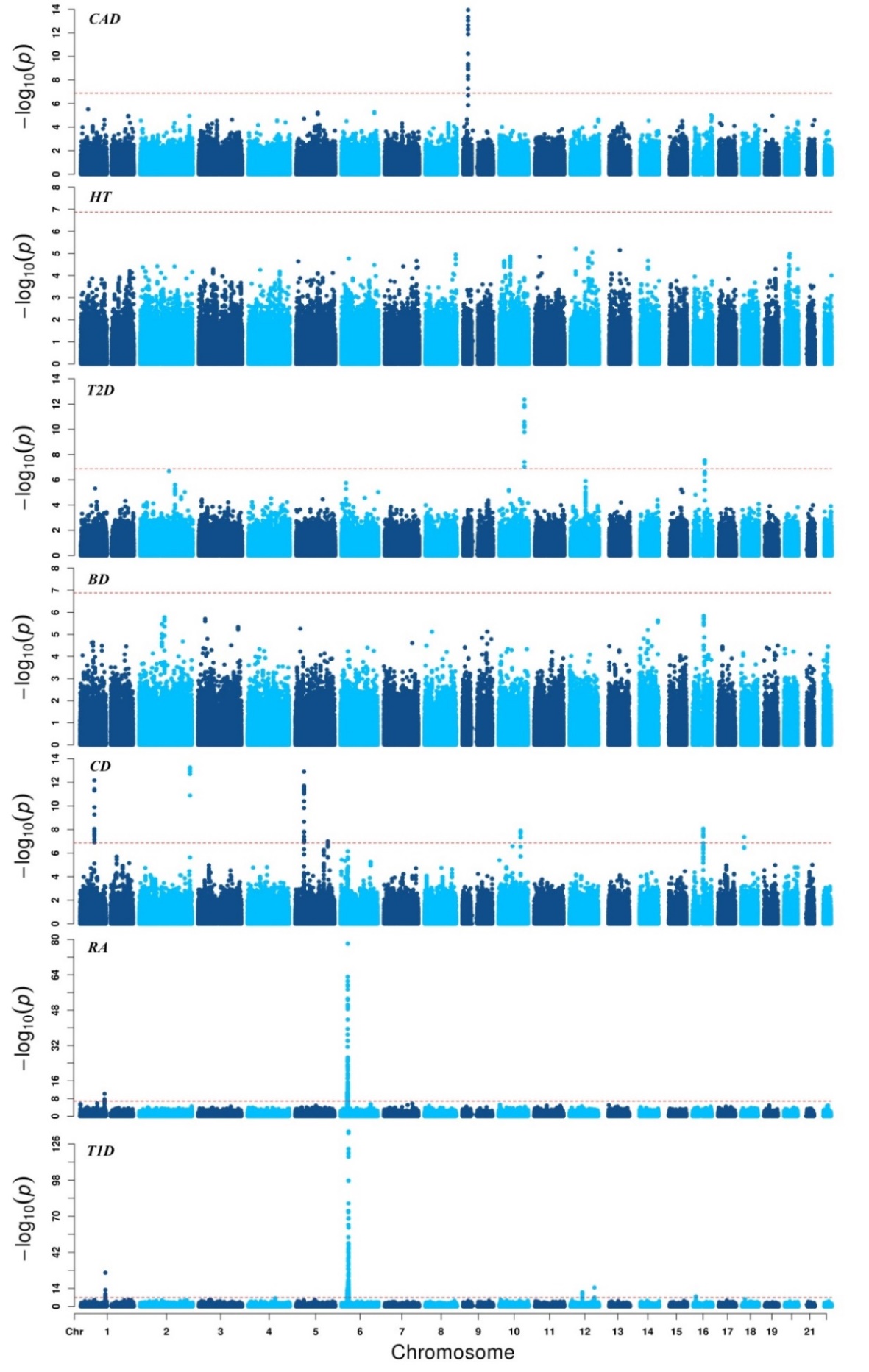


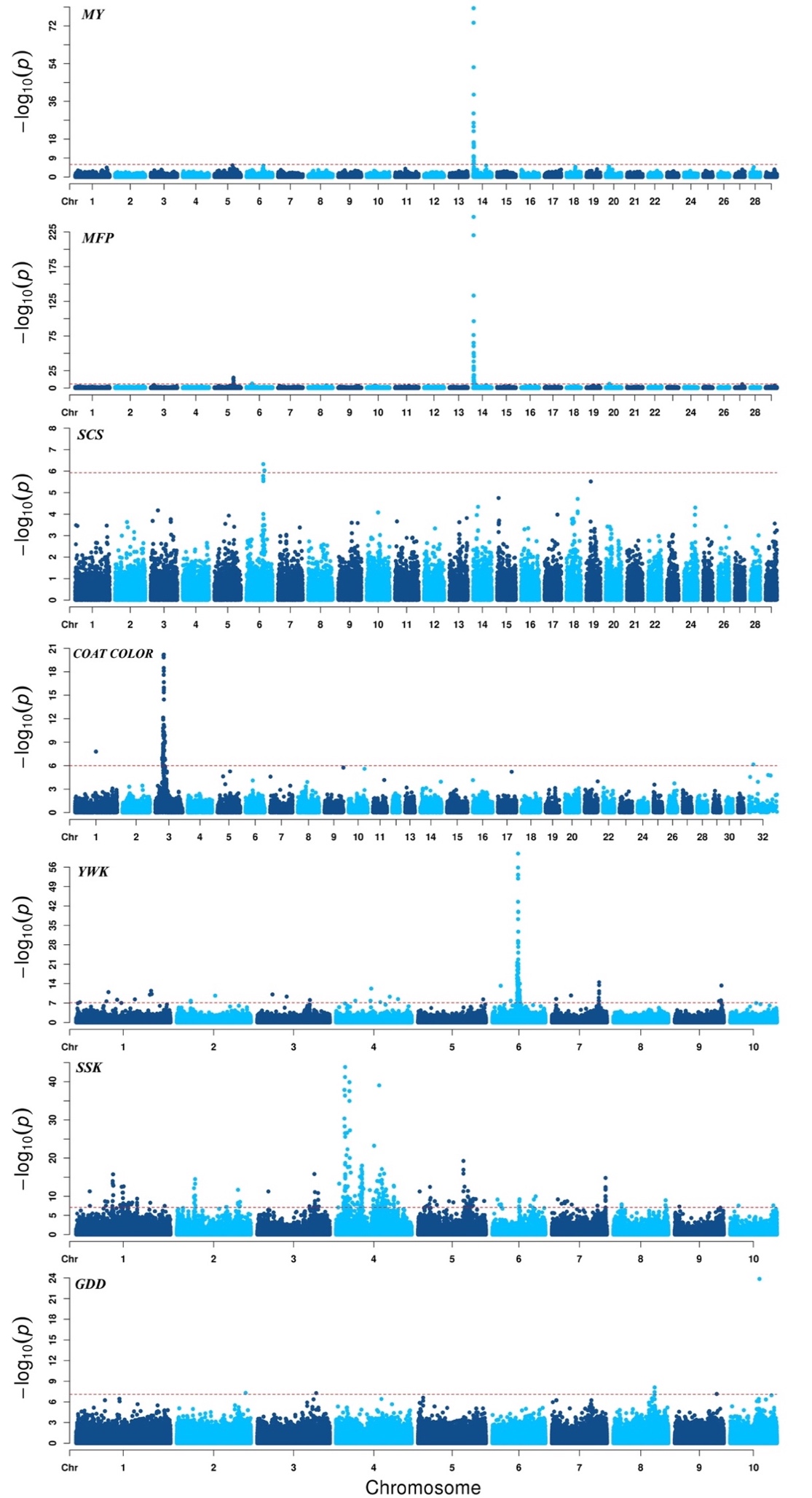


The trait name is marked on the top left of each figure.

Fig. S2. Prediction performances for LMM, BSLMM, BayesR, and KAML methods with different numbers of total MCMC iterations on three maize traits.


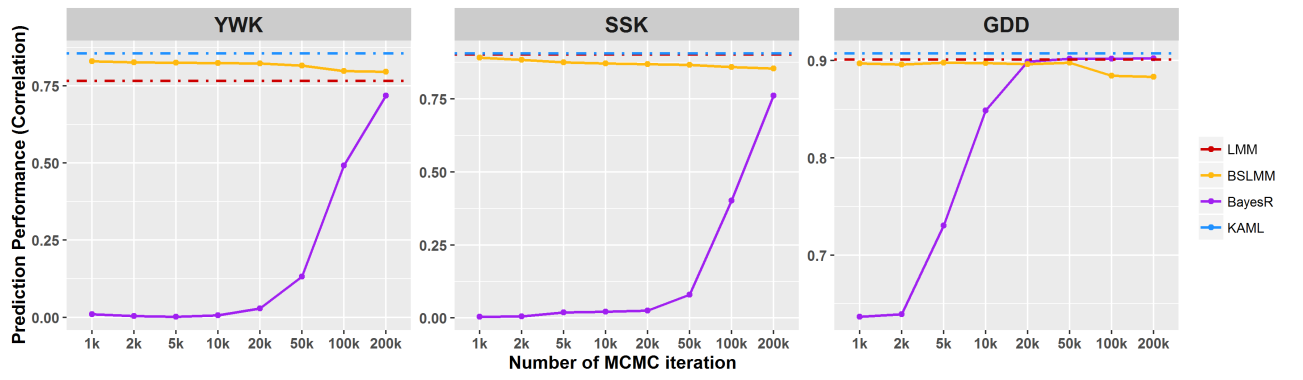


The average prediction accuracy value of 20 replicates is shown for each point. In each replicate, the dataset was randomly split into a reference subset that contained 80% of individuals and a validation subset that contained the remaining 20%. The number of burn-in iterations was set to 40% of the total number of iterations for both BSLMM and BayesR. The Y axis represents the prediction accuracy, and the X axis represents the total number of MCMC iterations.

Fig. S3. The calculating time (sec) of LMM, BSLMM, BayesR, and KAML methods with different numbers of total MCMC iterations on three maize traits.


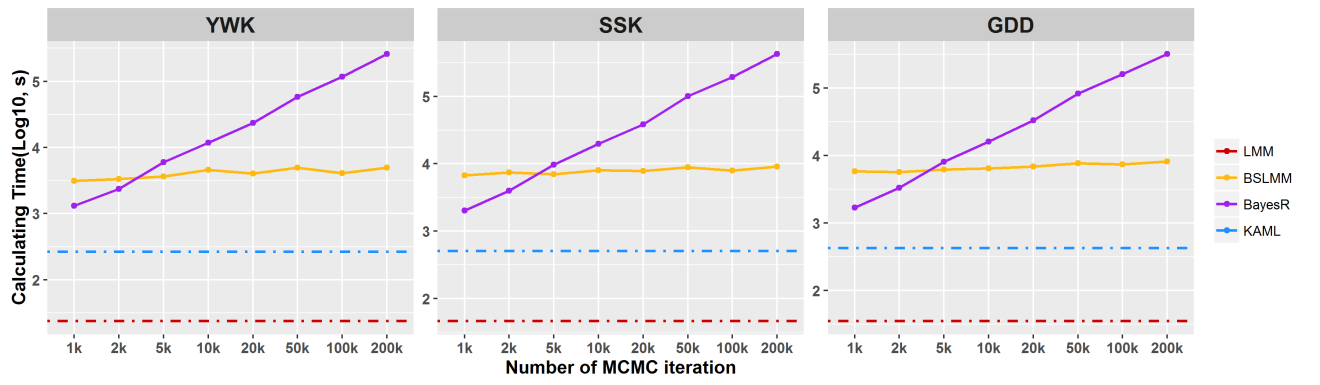


The average calculating time of 20 replicates in log_10_ is shown for each point. In each replicate, the dataset was randomly split into a reference subset that contained 80% of individuals, and a validation subset that contained the remaining 20%. The Y axis represents the calculating time, and the X axis represents the total number of MCMC iterations.
