# Supplementary material for: KAML: improving genomic prediction accuracy of complex traits using machine learning determined parameters
Source: Genome Biol. 2020 Jun 17;21:146. doi: 10.1186/s13059-020-02052-w (PMC7386246; doi:10.1186/s13059-020-02052-w)
Supplement: Supplementary file 2 — Additional file 2. Review history. [file 13059_2020_2052_MOESM2_ESM.docx]

Review History

**First round of review**

**Reviewer 1**

**Were you able to assess all statistics in the manuscript, including the appropriateness of statistical tests used?**

Yes: No additional statistical review required.

**Were you able to directly test the methods?**

Yes

**Comments to author:**

The manuscript describes software for genomic prediction. The software implements a method that provides improved performance and speed for genomic prediction. It does that by using cross-validation to select among a few different models and to optimize parameters for weighting a kinship matrix. It uses both simulated and real data sets from humans, cattle, horses, and maize to demonstrate the accuracy and speed of the method relative to GBLUP and selected Bayesian methods. While slower than GBLUP, it is demonstrated to be more accurate for oligogenic traits. The new method is considerably faster and as accurate as the Bayesian methods for the data sets analyzed. The idea of doing model selection among a few different linear models is a relatively simple approach, but it is one I have not seen proposed before for GWAS software and is surprisingly effective. Also, the software implements single step regression, a method which combines pedigree and genomic relationship matrices to calculate a kinship matrix. The authors have done a good job of describing and demonstrating the methods effectiveness. Given the widespread use of genomic prediction and selection in breeding programs, the new software should be of broad interest. The data sets used cover a wide range of data types and are an appropriate choice to evaluate the methods.

The biggest problem with the manuscript is that it contains many grammatical and word choice errors. The writing is poor enough in places to interfere with the understandability of the manuscript. An example includes line 290 which claims that false positive results might arise from "mystical relationships between individuals." The authors may have meant "cryptic relationships". The manuscript would need to be extensively edited for English usage before publication.

Specific problems,suggestions, and questions:
Line 146 seems to suggest that heritability is related to trait complexity. That is, that simpler traits are more more heritable than more complex ones. While there may be a tendency in that direction, that is not generally true and should be removed.
In the results section around line 288, cattle and maize traits are introduced and the traits abbreviated without explaining what the abbreviations stand for. The actual trait names need to be used here for understandability, even though they are given later in methods. As another example of language usage problems, traits are referred to as "quality traits" and "quantity traits". "Qualitative" and "quantitative" would be the proper terms.
In table 2, accuracy is measured as AUC (area under the curve). More detail needs to be provided as there are a couple of common curves used for this purpose. If it is the area under a ROC curve, it should be called AUROC. The methods section should include a description of how that was calculated.
In some cases, the manuscript mentions which model of the 5 model types was used by KAML, but it is not reported systematically for each of the simulated and real data sets. It would be a good idea to include that somewhere, perhaps in the tables reporting the accuracies.
In equation 5, no justification is given for the method used to weight SNPs. Explain how the weighting method was derived or chosen or provide a reference.
In line 567, "causal" not "casual".
In the SNP weighted optimization grid search how is A (accuracy) calculated?
For case control phenotypes, how was the phenotype modeled. Was it as 0 and 1 fit as a continuous variable? Similarly for horse coat color, which seems to be a categorical variable with 4 classes, how was the phenotype handled? That does not translate readily into a continuous variable. In other words, more detail needs to be provided describing how the categorical traits were analyzed.

**Reviewer 2**

**Were you able to assess all statistics in the manuscript, including the appropriateness of statistical tests used?**

Yes

**Were you able to directly test the methods?**

No

**Comments to author:**

Please consider the following questions:
- Are the methods appropriate to the aims of the study, are they well described, and are necessary controls included?
Yes.  
- Are the conclusions adequately supported by the data shown?
They are ok.
- Are sufficient details provided to allow replication and comparison with related analyses that may have been performed?
Yes.
- Is the method likely to be of broad utility? Is any software component easy to install and use? Please indicate briefly the novel features and/or advantages of the method, and/or please reference the relevant publications and which methods, if any, it should be compared with.
I doubt. I do not feel that the proposed method really advances the existing methods to a much higher level.
- Is the paper of broad interest to others in the field, or of outstanding interest to a broad audience of biologists?
Not really.


λ     Line 73.  "can't" should be "cannot".
λ      Lines 88-89. The first sentence is not a complete sentence.
λ     Lines 111-114. Splitting each data set into test and training sets should be repeated at least five times in order to investigate the stability of prediction results.  
λ     Line 128. "don't" could be " do not".
λ     Lines 145-150. Simulating SNP data sets may be unrealistic. Many people use real SNP datasets.
λ     Inspecting Tables 1 and 2 does not reveal that the proposed method is much better than the three competing methods.
λ     Line 505. Please chck the sentence.
λ     Line 514. Adding fixed effects can be regarded as at most being marginal.

**Authors Response**

**Point-by-point responses to the reviewers’ comments:**

Comment

*Response*

Responses to Reviewer #1:

(1) The manuscript describes software for genomic prediction. The software implements a method that provides improved performance and speed for genomic prediction. It does that by using cross-validation to select among a few different models and to optimize parameters for weighting a kinship matrix. It uses both simulated and real data sets from humans, cattle, horses, and maize to demonstrate the accuracy and speed of the method relative to GBLUP and selected Bayesian methods. While slower than GBLUP, it is demonstrated to be more accurate for oligogenic traits. The new method is considerably faster and as accurate as the Bayesian methods for the data sets analyzed. The idea of doing model selection among a few different linear models is a relatively simple approach, but it is one I have not seen proposed before for GWAS software and is surprisingly effective. Also, the software implements single step regression, a method which combines pedigree and genomic relationship matrices to calculate a kinship matrix. The authors have done a good job of describing and demonstrating the methods effectiveness. Given the widespread use of genomic prediction and selection in breeding programs, the new software should be of broad interest. The data sets used cover a wide range of data types and are an appropriate choice to evaluate the methods.

*Response: We sincerely thank you for your positive assessment, and we appreciate that you believe our method will be of broad interest to the community.*

(2) The biggest problem with the manuscript is that it contains many grammatical and word choice errors. The writing is poor enough in places to interfere with the understandability of the manuscript. An example includes line 290 which claims that false positive results might arise from "mystical relationships between individuals." The authors may have meant "cryptic relationships". The manuscript would need to be extensively edited for English usage before publication.

*Response: Thank you for pointing out this issue. We improved the understandability of the manuscript and highlighted all the changes. BTW, we corrected the ‘mystical relationships’ to ‘cryptic relationships’ based on your comments (Line 303).*

(3) Line 146 seems to suggest that heritability is related to trait complexity. That is, that simpler traits are more more heritable than more complex ones. While there may be a tendency in that direction, that is not generally true and should be removed.

*Response: Thank you for pointing out the issue, and we agree with you that the original sentence may lead to misunderstanding. We removed the original sentence following your suggestion; instead, we described the simulation scenarios as: “To be as realistic as possible, we simulated phenotypes of three scenarios in heritabilities of 0.2, 0.5, and 0.8 with different groups of SNPs corresponding to various genetic architectures that used the genotypes of the WTCCC1 dataset” (Line 147-150).*

(4) In the results section around line 288, cattle and maize traits are introduced and the traits abbreviated without explaining what the abbreviations stand for. The actual trait names need to be used here for understandability, even though they are given later in methods. As another example of language usage problems, traits are referred to as "quality traits" and "quantity traits". "Qualitative" and "quantitative" would be the proper terms.

*Response: Many thanks for pointing out the issue. We added the full actual name for each trait at the first occurrence of the abbreviation. We appreciate your kind reminder of the unprofessional terms, and all of them were corrected at corresponding positions (Line 166, 299, 690).*

(5) In table 2, accuracy is measured as AUC (area under the curve). More detail needs to be provided as there are a couple of common curves used for this purpose. If it is the area under a ROC curve, it should be called AUROC. The methods section should include a description of how that was calculated.

*Response: Thank you for your professional suggestion. Yes, we calculated the area under a ROC curve under the guidance of the paper published by Wray NR et al [1]. The method is defined as follows:*

$$AUROC=\frac{1}{N_{d'}}(\bar{r}_{d}-\frac{N_{d}}{2}-\frac{1}{2})$$

*where* $N_{d}$ *and* $N_{d'}$ *represent the numbers of diseased and not diseased individuals, respectively, and* $\bar{r}_{d}$ *is the mean rank of the diseased individuals. In the revised manuscript, we added the description of AUROC in the Method section (Line 608-613), and all the “AUC” were replaced by “AUROC”.*

(6) In some cases, the manuscript mentions which model of the 5 model types was used by KAML, but it is not reported systematically for each of the simulated and real data sets. It would be a good idea to include that somewhere, perhaps in the tables reporting the accuracies.

*Response: Thank you for your professional suggestion. We provide the model selection details for the real data analysis in the following table. And we have attached this table in Table S7 of the supplementary materials. For the traits simulated by us, the model selection details can be found in Table S2. For the public dataset simulated by the 16th QTL-MAS Workshop, there were no replications because the reference and predicted individuals were fixed, and the final confirmed model of KAML were all “pQ+Kw”, so we did not list them in the table, but mentioned it in the manuscript (Line 217-218). Below please find the table for the counts of selected models in the model parameter optimization procedure in datasets of multiple species.*

| *Species* | *Traits* | *KAML* | | | | |
| --- | --- | --- | --- | --- | --- | --- |
|  |  | *pQ* | *pQ+Kw* | *pQ+Ks* | *Kw* | *Ks* |
| *Human* | *cad* | *0* | *12* | *2* | *6* | *0* |
|  | *ht* | *0* | *0* | *0* | *13* | *7* |
|  | *t2d* | *0* | *13* | *0* | *7* | *0* |
|  | *bd* | *0* | *0* | *2* | *15* | *3* |
|  | *cd* | *0* | *20* | *0* | *0* | *0* |
|  | *ra* | *0* | *20* | *0* | *0* | *0* |
|  | *t1d* | *0* | *20* | *0* | *0* | *0* |
| *Cattle* | *mfp* | *0* | *20* | *0* | *0* | *0* |
|  | *my* | *0* | *20* | *0* | *0* | *0* |
|  | *scs* | *0* | *20* | *0* | *0* | *0* |
| *Horse* | *coat colour* | *18* | *1* | *1* | *0* | *0* |
| *Maize* | *ywk* | *0* | *20* | *0* | *0* | *0* |
|  | *ssk* | *0* | *9* | *0* | *11* | *0* |
|  | *gdd* | *0* | *20* | *0* | *0* | *0* |

*The pQ is the pseudo QTNs, Ks is the standard Kinship, and Kw is the SNP-weighted Kinship. The five models in KAML are (1) regular LMM (“Ks”); (2) LMM with SNP-weighted Kinship (“Kw”); (3) LMM with pseudo QTNs as covariates and standard Kinship (“pQ+Ks”); (4) LMM with pseudo QTNs as covariates and SNP-weighted Kinship (“pQ+Kw”); (5) Linear Model with pseudo QTNs as covariates (“pQ”).*

(7) In equation 5, no justification is given for the method used to weight SNPs. Explain how the weighting method was derived or chosen or provide a reference.

*Response: Thank you for pointing it out. The derivation of weighting SNPs in constructing Kinship matrix can be found in the paper published by Su et al. [2]. However, we proposed a more flexible and robust weighting strategy with fewer unknown parameters to be estimated. In the revised manuscript, we added some descriptions and cited the proper reference as well (Line 539-545).*

(8) In line 567, "causal" not "casual".

*Response: Sincerely thank you for your careful and patient review. We corrected it in the revised manuscript (Line 77, 441, 446, 593).*

(9) In the SNP weighted optimization grid search how is A (accuracy) calculated?

*Response: Thank you for the question. The accuracy in SNP weighted optimization grid search was calculated based on two categories of phenotypes. If the phenotype was defined as two levels (0 and 1), then KAML calculated the AUROC, otherwise, KAML considered it to be a quantitative trait and calculated the Pearson correlation. To make it clearer, we added this description in the revised manuscript (Line 604-608).*

(10) For case control phenotypes, how was the phenotype modeled. Was it as 0 and 1 fit as a continuous variable? Similarly, for horse coat color, which seems to be a categorical variable with 4 classes, how was the phenotype handled? That does not translate readily into a continuous variable. In other words, more detail needs to be provided describing how the categorical traits were analyzed.

*Response: We much appreciate your professional questions. Yes, the case control phenotype was fit as 0 and 1 when running all software as the previously published studies did [3, 4], and the predicted values can be treated as the probability of being the case. For horse coat colour, the provided phenotypes from the publishers were already coded as 1, 2, 3, 4 for corresponding colours [5], and it was described that the coat colour was divided into four categories by the shades of black based on three known coat colour loci that included MC1R (chestnut), STX17 (grey), and ASIP (agouti, black/bay). Therefore, we used the original phenotype as a continuous trait to run all software directly to make a fair comparison. To make it clear, we added these descriptions in the “Results” section (Line 317-321).*

Responses to Reviewer #2:

(1) Are the methods appropriate to the aims of the study, are they well described, and are necessary controls included? -- Yes.

Are the conclusions adequately supported by the data shown? -- They are ok.

Are sufficient details provided to allow replication and comparison with related analyses that may have been performed? -- Yes.

*Response: We would like to sincerely thank you for your positive assessments and confirmation of our work and effort.*

(2) Is the method likely to be of broad utility? Is any software component easy to install and use? Please indicate briefly the novel features and/or advantages of the method, and/or please reference the relevant publications and which methods, if any, it should be compared with. -- I doubt. I do not feel that the proposed method really advances the existing methods to a much higher level.

Is the paper of broad interest to others in the field, or of outstanding interest to a broad audience of biologists? – Not really.

*Response: Thank you for your comments. We would like to provide some explanations to address your concerns. As is well known, the genetic architecture of traits of animals, plants, and humans is far more complex than the fitting capacity of a model. Statistical genetics researchers have constantly been devoted to optimizing models to capture more genetic effects from observed traits, some of which have been widely used in academic research, e.g., BSLMM [3], BayesR [4], and DPR [6]. However, none of those models always performed the best across various traits, and they hardly achieved 1% or even 0.1% increase over others for prediction performance for very few traits. However, that 1% or 0.1% increase cannot be ignored because it can bring tremendous economic benefits, especially in a ‘pyramid’ breeding system. The unique advantage of KAML is that it includes various models relevant to qualitative and quantitative traits, and it can switch to one automatically using a machine learning strategy, which makes KAML more flexible and adaptable to various traits with a wide range of genetic architectures.*

*Moreover, the computational efficiency of a model is the key restriction in its realistic application, especially in animal and plant breeding programs. The estimated genetic breeding values need to be fed back to breeders in a short time to implement an earlier individual selection. But Bayesian models take days or even several weeks to obtain the unknown parameters, and this is the reason why Bayesian models are only used widely in academic research, but rarely applied to real breeding programs. Taking the advantages of efficient computing design and parallel computation, KAML is much more computationally efficient than Bayesian models without sacrifices in prediction accuracy, which further makes it more valuable for academic research or real breeding. From another point of view, the prediction accuracy of KAML would be significantly increased by handling a much larger dataset in a reasonable time compared with the advanced Bayesian models. Additionally, KAML can easily be integrated with the single step approach to make full use of both genomic and pedigree information.*

*With the increasing number of research publications for complex diseases, more QTLs and candidate genes will be identified by advanced statistical analysis; and integrating this information into prediction model would potentially improve the prediction accuracy. However, it is difficult to evaluate the reliability of prior knowledge except for independent molecular experiments, directly incorporating this information into the prediction model is of high risk. We discussed the potential advantages of KAML, which can provide a robust and efficient machine learning-based strategy to evaluate and use prior information, making KAML have the potential to be adapted to a wide gamut of applications in future genomic prediction or even omics prediction. Therefore, we are confident that KAML should be of outstanding interest to a broad audience of biologists.*

(3) Line 73. “can't” should be “cannot”. Line 128. "don't" could be “do not”

*Response: Sincerely thank you for your careful and patient review. We corrected it in the revised manuscript (Line 74, 130, 423, 455).*

(4) Lines 88-89. The first sentence is not a complete sentence.

*Response: Thank you for pointing out the issue. We corrected it as following “Inspired by the high prediction accuracy of a SNP-weighted strategy and efficient calculation of LMM, here we present a Kinship-Adjusted-Multiple-Loci (KAML) linear mixed model, which is a flexible modelling framework that generalizes the LMM to accommodate traits with various types of genetic architectures by incorporating pseudo QTNs (Quantitative Trait Nucleotides) as covariates and a SNP-weighted trait-specific Kinship as the variance-covariance assumption corresponding to the random effect term.” (Line 89-94).*

(5) Lines 111-114. Splitting each data set into test and training sets should be repeated at least five times in order to investigate the stability of prediction results.

*Response: Thank you for pointing out the issue, and we agree with you. For prediction assessment, total samples were split into two subsets, where 80% of the samples were used as a reference or training dataset and 20% were used as a validation or test dataset. The procedure was repeated at least 20 times in our study. This means that the values displayed in the manuscript were the average of 20 repeats. We are sorry that we didn’t declare it clearly at the beginning of “Results” section. We addressed the issue by adding a clear description based on your suggestion (Line 115-116).*

(6) Lines 145-150. Simulating SNP data sets may be unrealistic. Many people use real SNP datasets.

*Response: Thank you for raising this point, and we agree with you that the simulated SNP data sets are always unrealistic. In our study, we first demonstrated the flexibility of our model by the simulated traits on human dataset, then we compared our model with existing models on simulated dataset of public and real traits of humans, cattle, horses, and maize data sets. The only simulated SNP data set used in this study was from the 16th QTL-MAS Workshop. It includes a simulated SNP dataset, three traits with various genetic architectures, and complete pedigree information. Because the dataset was simulated by an authoritative organization and all the simulation details were clearly described, it had been widely used in previous published researches, including the domain of genome-wide associate study [7-9] and genomic prediction [10-12], making it more trustworthy, and the provided pedigree information can be perfectly utilized in our SSKAML for comparison, moreover, it is publicly accessible which can assure the results in our manuscript to be reproducible, thus we selected the dataset to verify the performance of KAML.*

*Following your suggestion, we simulated three traits in heritabilities of 0.2, 0.5, and 0.8 using the genotype of human dataset. We simulated the additive genetic effects of the traits that were controlled by 10 SNPs with large effects that were distributed as N(0,0.1), 1,000 SNPs with moderate effects that were distributed as N(0,0.01), and 10,000 SNPs with polygenic effects that followed a normal distribution with a mean value of 0 and a variance of 0.005. Following previous studies [13], we implemented the experiments on chromosome 1 with 20 replicates for each scenario to save time. The reported prediction accuracies are shown in the following table, we found that KAML significantly outperformed LMM for all scenarios, and performed slightly better than Bayesian models. We have attached this table in Table S3 of the supplementary materials and added descriptions accordingly in the revised manuscript following your suggestion (Line 195-207).*

| *Heritability* | *Methods* | | | |
| --- | --- | --- | --- | --- |
|  | *LMM* | *BSLMM* | *BayesR* | *KAML* |
| *0.2* | *0.4312* | *0.5632* | *0.5510* | *0.5680* |
| *0.5* | *0.5980* | *0.6815* | *0.6724* | *0.6814* |
| *0.8* | *0.7167* | *0.7722* | *0.7680* | *0.7728* |
| *Average* | *0.5820* | *0.6723* | *0.6638* | *0.6741* |

*Prediction accuracy was measured using Pearson correlation coefficients between the predicted values and the simulated additive genetic effect values. The average Pearson correlation coefficients of each scenario are shown on the basis of 20 replicates in the table*

(7) Inspecting Tables 1 and 2 does not reveal that the proposed method is much better than the three competing methods.

*Response: Thank you for raising this point. With respect to the prediction accuracy results shown in Tables 1 and 2, KAML outperformed LMM, BSLMM, and BayesR in eight, seven, and six of the ten traits, respectively. As described in the response to question (2), on the one hand, the genetic architecture of traits is far more complex than the fitting capacity of a model, 1% or even 0.1% increase on prediction accuracy is a hard step for previously published models. On the other hand, the prediction accuracy is always contrary to the computational efficiency for the existing models, advanced Bayesian models usually hold a more complex hypothesis that results in an increase in computing time. KAML is several hundred times faster than Bayesian models, and it generates similar or even slightly higher prediction accuracy. Considering the prediction accuracy performance in data of multiple species, especially maize, KAML is the only method that provides efficient, robust, and accurate genomic prediction.*

(8) Line 505. Please check the sentence.

*Response: Thank you for pointing out the issue. We have corrected the grammatical problem and rewritten the math formula (Line 528).*

(9) Line 514. Adding fixed effects can be regarded as at most being marginal.

*Response: Much appreciate for your professional suggestions. Following your suggestion, we replaced all the terms “fixed effects” with “covariates” in the revised manuscript.*

*We sincerely thank the reviewer #1 and reviewer #2 again for the helpful criticism and professional comments. We look forward to hearing from you regarding our submission and to respond to any further questions and comments you may have.*

*Reference*

*1.	Wray NR, Yang J, Goddard ME, Visscher PM: The genetic interpretation of area under the ROC curve in genomic profiling. PLoS Genet 2010, 6:e1000864.*

*2.	Su G, Christensen OF, Janss L, Lund MS: Comparison of genomic predictions using genomic relationship matrices built with different weighting factors to account for locus-specific variances. J Dairy Sci 2014, 97:6547-6559.*

*3.	Zhou X, Carbonetto P, Stephens M: Polygenic modeling with bayesian sparse linear mixed models. PLoS Genet 2013, 9:e1003264.*

*4.	Moser G, Lee SH, Hayes BJ, Goddard ME, Wray NR, Visscher PM: Simultaneous discovery, estimation and prediction analysis of complex traits using a bayesian mixture model. PLoS Genet 2015, 11:e1004969.*

*5.	McCue ME, Bannasch DL, Petersen JL, Gurr J, Bailey E, Binns MM, Distl O, Guerin G, Hasegawa T, Hill EW, et al: A high density SNP array for the domestic horse and extant Perissodactyla: utility for association mapping, genetic diversity, and phylogeny studies. PLoS Genet 2012, 8:e1002451.*

*6.	Zeng P, Zhou X: Non-parametric genetic prediction of complex traits with latent Dirichlet process regression models. Nat Commun 2017, 8:456.*

*7.	Li H, Su G, Jiang L, Bao Z: An efficient unified model for genome-wide association studies and genomic selection. Genet Sel Evol 2017, 49:64.*

*8.	Grosse-Brinkhaus C, Bergfelder S, Tholen E: Genome wide association analysis of the QTL MAS 2012 data investigating pleiotropy. BMC Proc 2014, 8:S2.*

*9.	Neuditschko M, Raadsma HW, Khatkar MS, Jonas E, Steinig EJ, Flury C, Signer-Hasler H, Frischknecht M, von Niederhausern R, Leeb T, Rieder S: Identification of key contributors in complex population structures. PLoS One 2017, 12:e0177638.*

*10.	da Cruz Pereira GM, de Paula Ribeiro A, Martins Filho S: Genomic prediction of ordinal traits via application of the BLASSO and BayesCπ methods in simulated data. Multi-Science Journal 2020, 3:1-7.*

*11.	Song H, Li L, Zhang Q, Zhang S, Ding X: Accuracy and bias of genomic prediction with different de-regression methods. Animal 2018, 12:1111-1117.*

*12.	Silva FF, Jerez EAZ, de Resende MDV, Viana JMS, Azevedo CF, Lopes PS, Nascimento M, de Lima RO, Guimarães SEF: Bayesian model combining linkage and linkage disequilibrium analysis for low density-based genomic selection in animal breeding. Journal of applied animal research 2018, 46:873-878.*

*13.	Lloyd-Jones LR, Zeng J, Sidorenko J, Yengo L, Moser G, Kemper KE, Wang H, Zheng Z, Magi R, Esko T, et al: Improved polygenic prediction by Bayesian multiple regression on summary statistics. Nat Commun 2019, 10:5086.*

**Second round of review**

**Reviewer 1**

The authors' revision did an excellent job of addressing my original concerns. I did notice that is there is one paragraph in the methods section starting at line 579 that was apparentally missed by the language editor, who otherwise did a fine job. The first sentence of that paragraph is poorly worded and hard to follow. Also the paragraph contains two incomplete sentences and one run-on sentence.
